# Stability through plasticity: Finding robust memories through representational drift

**DOI:** 10.1073/pnas.2500077122

**Published:** 2025-11-07

**Authors:** Maanasa Natrajan, James E. Fitzgerald

**Affiliations:** ^a^Department of Neurobiology, Northwestern University, Evanston, IL 60208; ^b^Solomon H. Snyder Department of Neuroscience, Johns Hopkins University, Baltimore, MD 21205; ^c^Janelia Research Campus, HHMI, Ashburn, VA 20147; ^d^NSF-Simons National Institute for Theory and Mathematics in Biology, Chicago, IL 60611; ^e^Department of Physics and Astronomy, Northwestern University, Evanston, IL 60208; ^f^Department of Engineering Sciences and Applied Mathematics, Northwestern University, Evanston, IL 60208

**Keywords:** representational drift, solution space, robustness, sparsity, continual learning

## Abstract

Memories are believed to be stored in synapses and retrieved by reactivating neural ensembles. Learning alters synaptic weights, which can interfere with previously stored memories that share the same synapses, creating a trade-off between plasticity and stability. Interestingly, neural representations change even in stable environments, without apparent learning or forgetting—a phenomenon known as representational drift. Theoretical studies have suggested that multiple neural representations can correspond to a memory, with postlearning exploration of these representation solutions driving drift. However, it remains unclear whether representations explored through drift differ from those learned or offer unique advantages. Here, we show that representational drift uncovers noise-robust representations that are otherwise difficult to learn. We first define the nonlinear solution space manifold of synaptic weights for fixed input–output mappings, which allows us to disentangle drift from learning and forgetting and simulate drift as diffusion within this manifold. Solutions explored by drift have many inactive and saturated neurons, making them robust to weight perturbations due to noise or continual learning. Such solutions are prevalent and entropically favored by drift, but their lack of gradients makes them difficult to learn and nonconducive to future learning. To overcome this, we introduce an allocation procedure that selectively shifts representations for new stimuli into a learning-conducive regime. By combining allocation with drift, we resolve the trade-off between learnability and robustness.

## Significance

Although synaptic and neuronal changes are often associated with learning, many such changes occur without learning or forgetting in a phenomenon known as representational drift. Previous studies suggest that multiple synaptic configurations can generate the same neuronal pattern and multiple neuronal patterns can store the same memory, allowing the brain to explore different configurations for a memory. Here, we show that some of these configurations are robust to weight changes while others facilitate learning. Importantly, robust solutions are more common, making them naturally favored by random representational drift. Although no single configuration is both robust and learning-optimal, we show that representational drift, combined with an allocation procedure, enables the system to dynamically shift between robust and learning-conducive regimes as needed.

Cognition and behavior are thought to arise from neural activity patterns, leading many to equate brain functions with specific activity patterns, also called neural representations. For example, perhaps our sense of where we are amounts to which “place cells” fire in the hippocampus. Memory could in turn be the reactivation of neural activity patterns corresponding to past experiences, with Hebbian assemblies and memory engrams being prominent examples (1–3). However, recent data suggest that the mapping between neural representations and brain function is not one-to-one. In the hippocampus, which neurons represent any given place changes over time (4–10). Such shifts in neural representations are termed “representational drift” (11, 12), because they appear to be devoid of discernible learning, forgetting, or behavioral changes. This intriguing phenomenon raises questions about the neural substrates of experience and its memory.

Relatively clear hypotheses exist for how neural representations could drift without disrupting cognition or behavior. In machine learning, researchers train artificial neural networks to generate desired input–output mappings, and neurons in intermediate layers can adopt any activity pattern that yields the correct output. The fixed inputs and outputs somewhat anchor the intermediate representations, but many possibilities remain. Drift can thus be modeled as the random exploration of intermediate representations that produce the same outputs (13–17). In biology, sensory and motor representations rigidly tie brain activity to the external world, and the brain’s cognitive functions may impose additional constraints. Yet it is unlikely that these anchors remove all the redundancies. Supporting this view, representational drift is seen across many brain regions, including the hippocampus (6), posterior parietal cortex (18), prelimbic cortex (19), visual cortex (20), somatosensory cortex (21), and piriform cortex (22). However, drift seems to spare certain dimensions of neural activity (20, 23–28), pointing to low-dimensional substrates that might stably encode sensory, behavioral, and cognitive variables.

Drift may be a result of noisy learning systems. Researchers have proposed that neural representations underlying memories are prone to degradation from factors like intrinsic noise and continual learning (29, 30). Error-corrective processes could compensate, but it is likely that these will uncover a distinct representation rather than revert to the previous version, resulting in representational drift (15, 17, 31).

Assuming that drift explores representations encoding the same memory content, a fundamental question arises. How might representations uncovered through drift differ from those initially learned? Moreover, could drift-induced representations offer advantages over the original learned solutions? Here, we propose that drift benefits neural systems by helping them find noise-robust solutions. Our findings reveal that sparsely engaged representations, characterized by many inactive and saturated neurons, are more prevalent than densely engaged ones with fewer such neurons. Thus, random exploration of weight solution space favors sparse representations for entropic reasons. Importantly, these sparse representations are more robust to weight perturbations, which may occur due to noise or learning. However, these robust solutions are hard to learn, suggesting that drift may play a critical role in finding these solutions. Thus, our results suggest that representational drift is not merely a byproduct of noise and continuous learning. Rather, it can actively help maintain memories in the face of noise and continual learning.

## Results

### Theoretical Framework For Modeling Representational Drift.

**Fig. 1.**
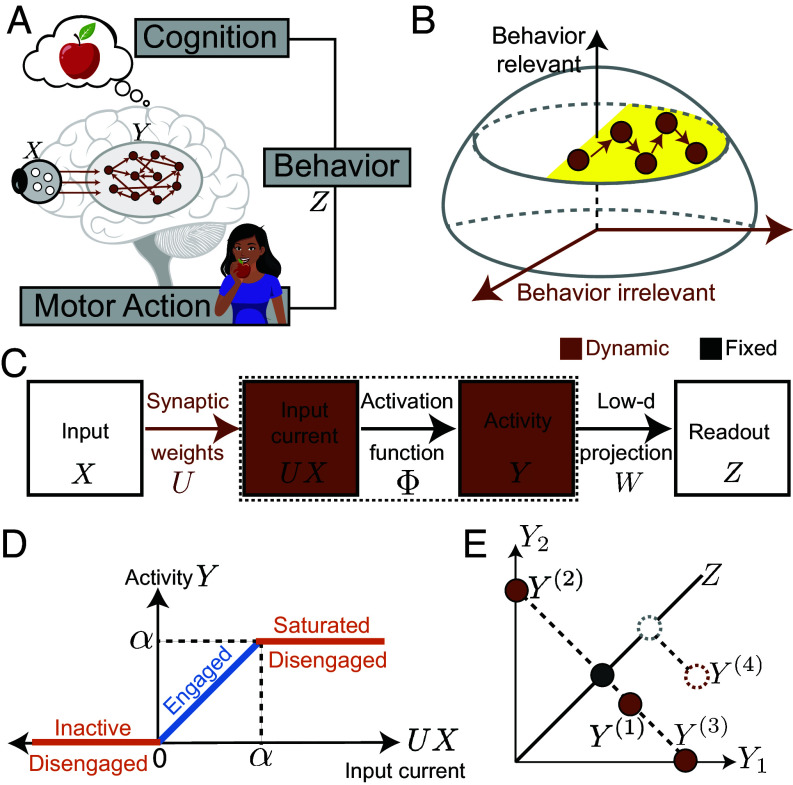
Theoretical framework for modeling representational drift. (*A*) Neural representation Y is generated from inputs X and determines behavioral readouts Z. (*B*) Illustration of a solution space (in yellow) of weights that generate the desired readout and drift as a bounded diffusion restricted to the solution space. (*C*) Series of transformations that lead from input (X) to readout (Z), with components that can change during drift in brown and fixed components in black. Elements within the dashed border correspond to neurons in the analyzed representation (Y). (*D*) Clipped-threshold-linear activation function that relates the input current (UX) to neural activity (Y). (*E*) Schematic showing readout (Z) as a low-dimensional projection of representation (Y), showing how multiple representations produce the same readout (solid circles).

We conceptualize representational drift as the exploration of neural network states that result in the same behavior, by which we mean both cognitive processes and control of the body. The neural mechanisms underlying behavior are incompletely understood, so we model behavior as an abstract function, Z, of neural activity, Y (Fig. 1*A*). For example, if Z represents the visual perception of an apple, Y could denote the firing rates of visual thalamus neurons, and Z(Y) would be a complicated nonlinear function. Alternatively, if Y represents activity in the high-level visual cortex, Z(Y) might be a simple linear readout. We assume that neural activity Y is mechanistically determined by feed-forward drive from an input population, X. For example, X might be retinal ganglion cells if Y is thalamus. We ensure finite synaptic weights by imposing a weight norm bound for each postsynaptic neuron (*SI Appendix*, *Methods*). We also assume that the activation function relating input currents to firing rates, Φ, has finite activation and saturation thresholds. For instance, a neuron has a firing rate of zero when the input current is below its activation threshold, and a neuron’s refractory period imposes a limit on its firing rate. We refer to neurons with intermediate activity as “engaged,” as their activity changes with variations in input. In contrast, neurons that are either inactive or saturated do not alter their activity in response to input changes, and we refer to them as “disengaged.” We focus on a single drifting population by assuming stable X and Z(Y). Representational drift in Y results from changing synaptic weights, and we model it as diffusion in the solution space of synaptic weights that generate specified X to Z mappings (Fig. 1*B*). Though we allow all neurons to drift, we can also extend our framework to include a stable subpopulation in Y (*SI Appendix*, section 1C and Fig. S3). We assume that the number of X to Z mappings, P, does not exceed the number of neurons providing input to Y. This allows us to locally construct a natural coordinate system for the solution space manifold (32, 33).

Most of our analyses will focus on a model variant that makes a few additional assumptions (Fig. 1*C*). First, we model the firing rate nonlinearity as a clipped-threshold-linear function (Fig. 1*D*). This implies that neurons remain inactive when the input current is below 0, become saturated when the input current exceeds α, and linearly increase their firing rates in between. This function makes it straightforward to specify the solution space manifold, but the solution space can also be analyzed for other activation functions (*SI Appendix*, section 1A). We include saturation because both inactive and saturated neurons will contribute similarly to limiting learnability and enhancing robustness—two central themes of this work. However, we also simulated biologically realistic scenarios where neurons rarely reach saturation and found similar results (*SI Appendix*, section 3B and Fig. S8). Second, we assume that the behavioral readout Z is a one-dimensional linear projection of Y (Fig. 1*E*). Thus, we can specify Z through readout weights, W, but these weights are abstract and need not correspond to synaptic weights. This assumption is inspired by the empirical observation that behaviorally relevant information can often be linearly decoded from neural activity (34, 35). Yet, nonlinear readouts may help understand drift in specific areas of interest (22–24, 36).

### Intuitive Summary of Framework and Main Results.

Humans and other animals exhibit a diverse array of behaviors, ranging from navigation to communication, generated in response to sensory cues and shaped by past experiences. For example, to reach their lab a student might use a restaurant as a landmark to turn right and a library as a cue to turn left (37). Similarly, a monkey might emit a particular pitch vocalization to alert others of a leopard, while using other sounds for other predators (38). In each case, the behavior is a low-dimensional output represented and generated by collective neural activity.

**Fig. 2.**
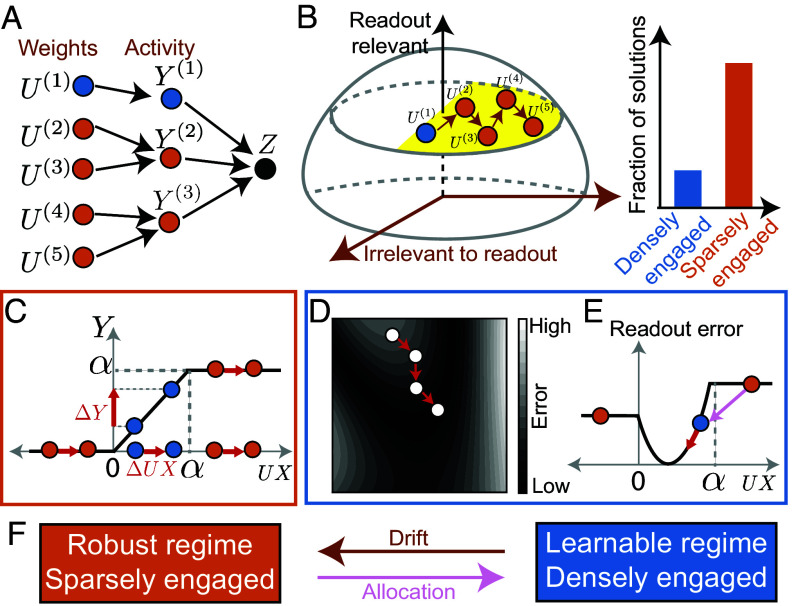
An intuitive summary: drift as diffusion in the solution space improves robustness at the cost of learnability. (*A*) An example mapping between synaptic configurations (U), neural representations (Y), and readout (Z), with densely engaged solutions in blue and sparsely engaged solutions in orange. (*B*) Schematic indicating that sparsely engaged solutions may be more prevalent within the solution space, so unbiased exploration in the weight solution space (in yellow) leads to sparsely engaged solutions. (*C*) Illustration that disengaged neurons (inactive or saturated) enhance robustness to changes in input currents. (*D*) Diagram showing that learning requires movement in a direction opposite to the error gradient. (*E*) Illustration that engaged neurons provide gradient information, enabling learning. (*F*) Illustration that drift along with allocation can overcome learnability–robustness trade-off.

What ultimately matters is not the neural representation or synaptic weights, but generating the correct behavior. For example, the student needs to make the correct turns to reach their lab, and the monkey must produce the right vocalization to warn of predators. Although the system is constrained by these demands, if behavior Z is low-dimensional compared to the neural representation Y, many representations, and in turn many synaptic weights U, may produce the same behavior (Fig. 2*A*) (11, 13). This implies a synaptic weight solution space that achieves the same behavior (Fig. 1*B*). In this paper, we will develop a framework for locally defining this solution space manifold and simulate drift as diffusion within it.

Diffusion in the solution space of synaptic weights implies that the system is equally likely to occupy any weight solution at a given time, so understanding representational drift requires a statistical description of the solution space. We first note that unbiased sampling of synaptic weight solutions can lead to biased sampling at the representation level. All nonpositive input currents result in an inactive neuron, all input currents exceeding the saturation threshold produce a saturated neuron, but a single input current leads a neuron to be engaged with a specific activity level (Fig. 1*D*). We will show that this generates dimensions in which synaptic weights and input currents can change without changing the representation, and the number of such dimensions is set by the number of disengaged (inactive or saturated) neurons. If not for the weight norm bound, the system could diffuse infinitely far into these dimensions. When the weight norm bound is large, this leads sparsely engaged representations to be more common (Fig. 2 *A* and *B*). Thus, we will find that representational drift can entropically favor sparsely engaged representations (Fig. 2*B*). For a more concrete explanation using a simple toy example see *SI Appendix* section 2A and Fig. S4. Depending on the choice of bounds, solutions with different sparsity levels can become more prevalent, an effect that can be both analytically understood and numerically verified (*SI Appendix*, section 2B and Fig. S5). In particular, for certain bias values and a small weight norm bound, drift favors sparsely active representations with many inactive neurons (*SI Appendix*, section 3B).

Although sparsely and densely engaged solutions produce the same behaviors, sparsely engaged representations favored by drift are advantageous. Returning to the example of a student navigating, consider two scenarios. First, the library used as a landmark becomes partially obscured, slightly altering the sensory input X. Alternatively, the same neural circuit is used to learn the route to a coffee shop, leading to changes in the weights U. In either case, the input current UX experiences a perturbation, but the student must make the correct turns. Neural representations that produce the same behavioral output can respond quite differently to these perturbations. Disengaged neurons are robust to small changes in input currents because their activity remains constant unless the thresholds are crossed (Fig. 2*C*). In contrast, engaged neurons are sensitive to changes in input currents, which influence their activity and potentially alter the behavioral output. Thus, disengaged neurons contribute to robustness against perturbations, making sparsely engaged representations more robust. We analytically compute the expected robustness as a function of the fraction of disengaged neurons, and this prediction closely matches the robustness observed in our simulations of representational drift, suggesting that drift improves robustness by discovering sparsely engaged solutions (*SI Appendix*, section 2C and Fig. S6).

This robustness comes at a cost. While the exact plasticity rules that govern synaptic changes remain unclear, learning requires that synaptic modifications lead to performance improvements (Fig. 2*D*). For example, if a student typically goes straight upon seeing the library, but a change in synaptic weights causes them to turn right instead, they might reach their lab more quickly, leading to better performance. However, only engaged neurons are responsive to these synaptic weight changes and thereby able to affect neural activity and behavior (Fig. 2*E*). Engaged neurons, but not disengaged ones, provide feedback on performance changes. The very property that makes disengaged neurons good for robustness—resistance to perturbations—makes them less useful for learning.

This reveals the learnability–robustness trade-off. Sparsely engaged representations are good for maintaining memories, while densely engaged ones are good for learning (for concrete examples of solutions spanning the learnability–robustness spectrum, see *SI Appendix*, section 2A and Fig. S4). We will see that drift naturally leads to sparsely engaged representations, facilitating a transition from learning-conducive states to more robust ones (Fig. 2*F*). However, to support new learning, we will also need a mechanism that shifts representations back to the learnable regime. We will thus introduce an allocation process that makes neurons engaged for new input conditions, creating densely engaged representations conducive to learning without disrupting stored memories. By combining drift with allocation, we propose a dynamic solution to the learnability–robustness trade-off in which the brain could maintain both adaptability for learning and robustness for memory retention.

### Characterization of the Solution Space and Restricted Diffusion.

To model representational drift, we first develop a mathematical approach to simulate diffusion restricted to the solution space. We consider an $N_{y}$-dimensional representation that has to generate an $N_{z}$-dimensional readout from its $N_{x}$-dimensional input under P different input conditions. The readout is modeled as a linear projection of the representation. The input–output transformation of the system is thus:

[1] $$\begin{array}{c} z_{i}=\sum_{j=1}^{N_{y}}W_{i,j}y_{j}=\sum_{j=1}^{N_{y}}W_{i,j}\Phi \left(\sum_{k=1}^{N_{x}}U_{j,k}x_{k}+b_{j}\right), \end{array}$$

where z is an $N_{z}$-vector defining the readout, W is a $N_{z}\times N_{y}$ linear projection matrix, y is an $N_{y}$-vector for the representation, U is an $N_{y}\times N_{x}$ matrix of synaptic weights, x and b are $N_{x}$-vectors of inputs and biases, and Φ is the clipped-threshold-linear activation function (with saturation threshold α) relating input current to activity (Fig. 1*D*)

[2] $$\begin{array}{c} \Phi \left(v\right)=\left\{\begin{array}{cc} 0 & \text{if}\;v\leq 0\\ v & \text{if}\;0<v<\alpha \\ \alpha & \text{if}\;v\geq \alpha \end{array}\right.. \end{array}$$

The constraints imposed by the P input–output mappings are

[3] $$\begin{array}{c} Z_{i,\mu }=\sum_{j=1}^{N_{y}}W_{i,j}Y_{j,\mu }=\sum_{j=1}^{N_{y}}W_{i,j}\Phi \left(\sum_{k=1}^{N_{x}}U_{j,k}X_{k,\mu }+B_{j,\mu }\right), \end{array}$$

where μ=1,⋯,P indexes the conditions, Z is an $N_{z}\times P$ matrix of the required readouts, Y is an $N_{y}\times P$ matrix of representations across P conditions, X is an $N_{x}\times P$ matrix of inputs, and B is a $N_{y}\times P$ matrix formed by repeating bias vector b as columns P times. Note that Eq. **3** is simply $Z=W\Phi \left(UX+B\right)$ in matrix notation.

While here we consider a clipped-threshold linear activation function, our proposed solution space manifold setup can be easily applied to a broader class of nonlinear activation functions (*SI Appendix*, section 1A). The choice of bias, activation function, and constraints influences the prevalence of different types of solutions (*SI Appendix*, section 2B). As we will see later, zero bias and large weight norm bounds tend to favor sparsely engaged solutions, characterized by many inactive and saturated neurons. Negative bias and small weight norm bounds favor sparsely active solutions, with predominantly inactive neurons (*SI Appendix*, section 3B and Fig. S8), which exhibit similar robustness advantages and limitations in learnability.

**Fig. 3.**
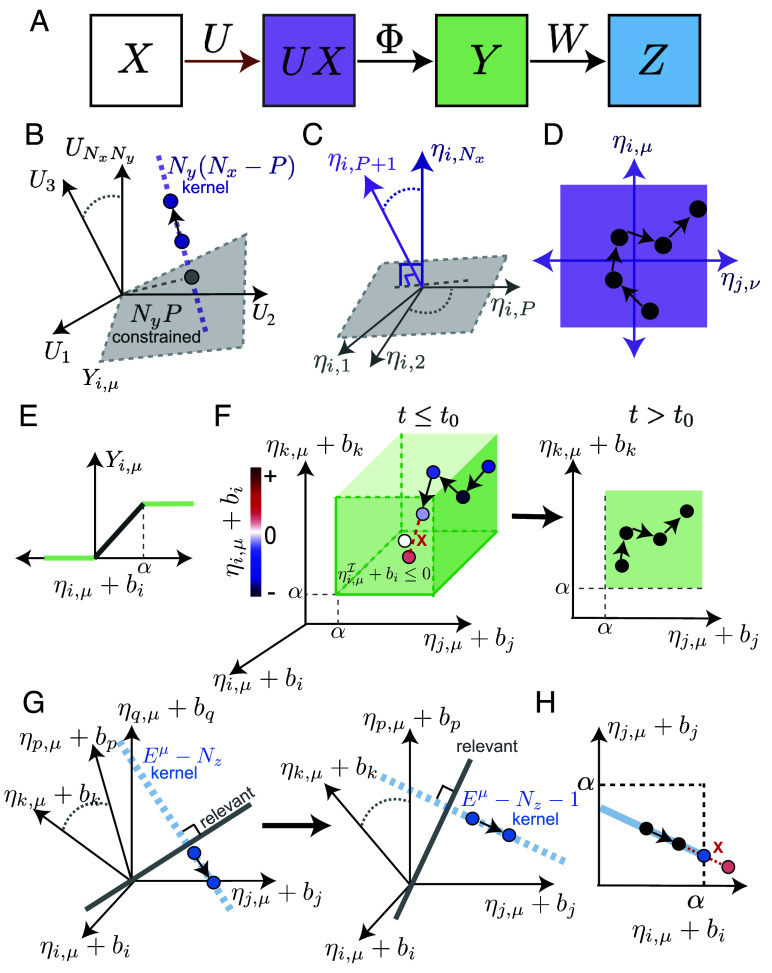
Characterization of the solution space and restricted diffusion. (*A*) Two linear transformations, U and W, and a nonlinear transformation, Φ, together generate readout Z from input X via representation Y. Changing U (brown arrow) can result in fixed UX (purple, *B*–*D*), dynamic UX but fixed Y (green, *E* and *F*), or dynamic Y but fixed Z (blue, *G* and *H*). (*B*) Schematic depicting the $N_{y}P$-dimensional constrained subspace (gray), into which projections fully determine UX, and the $N_{y}\left(N_{x}-P\right)$-dimensional kernel of X (purple), in which unconstrained changes can occur. (*C*) Schematic of η-coordinates, which provide a convenient coordinate system on the synaptic weights by separating constrained (gray) and unconstrained (purple) dimensions for each neuron i. (*D*) Schematic of weight diffusion in unconstrained dimensions, where μ,ν>P, and i,j index arbitrary postsynaptic neurons. (*E*) The clipped-threshold-linear activation function provides flexibility by producing the same output for many inputs. $b_{i}$ is the bias of the ith neuron and $\eta_{i,\mu }+b_{i}$ represents the input current of neuron i under condition μ. This flexibility is confined to one side of the threshold, so we refer to its dimensions as semiconstrained. (*F*) Semiconstrained dimensions change over time. (*Left*) Diffusion in the semiconstrained subspace, where μ indexes the condition, neuron i is inactive, and neurons j and neuron k are saturated. At time $t_{0}$, a random change causes neuron i to cross the activation threshold and become engaged. $\eta_{i\mu }+b_{i}$ is set to 0, and neuron i is shifted to engaged set. (*Right*) After $t_{0}$, the number of semiconstrained dimensions is reduced and the semiconstrained diffusion continues. (*G*) Representational drift dimensions change over time and involve multiple neurons. Visualization of the $E^{\mu }$-dimensional engaged subspace for condition μ≤P, with $E^{\mu }=\left\{i,j,k,\cdots ,p,q\right\}$. The engaged submatrix $W^{\mu }$ defines a $N_{z}$-dimensional relevant projection (gray), leaving $E^{\mu }-N_{z}$ flexible dimensions in its kernel (blue). (*Left*) Changes in these dimensions cause drift, and neuron q eventually crosses the activity threshold and becomes inactive at time $t_{0}$. (*Right*) Later, the flexible dimensions are confined to the kernel of the new engaged submatrix, which has $E^{\mu ,t_{+}}-N_{z}=E^{\mu ,t_{-}}-N_{z}-1$ dimensions, where $t_{-}$ and $t_{+}$ denote times shortly before and shortly after $t_{0}$. (*H*) Schematic illustrating how changes are rescaled to keep drift in the kernel of $W^{\mu }$ and prevent threshold crossings, where $i,j\in E^{\mu }$.

We model representational drift as diffusion in the solution space of weights U that satisfy Eq. **3** for μ=1,⋯,P. Specifically, we characterize three kinds of flexibility (Fig. 3*A*): 1) changes in synaptic weights U that maintain the input currents UX; 2) changes in input currents that maintain the representation Y; and 3) changes in the representation Y that maintain the readout Z. The first two kinds of flexibility entail synaptic weight changes concealed at the representation level, so we call these covert exploration dimensions. The third kind alters the representation, so we call these drift dimensions.

The first type of flexibility arises when the number of input neurons exceeds the number of conditions, $N_{x}>P$, because this implies a many-to-one mapping from weights to input currents. In particular, changes in U limited to the $N_{y}\left(N_{x}-P\right)$-dimensional kernel of X do not alter the input current UX (Fig. 3*B*). We call these unconstrained dimensions because there is complete freedom in these dimensions. Unconstrained dimensions can be separated out when we consider U in a coordinate system designed to manifest the activity-dependent constraints (Fig. 3*C*) (32, 33). We introduce a basis transformation matrix, $X_{\mathrm{ext}}$, where $X_{\mathrm{ext}}$ is an $N_{x}\times N_{x}$ full-rank matrix composed of X and $N_{x}-P$ columns that span the kernel of X. The new coordinates are given by

[4] $$\begin{array}{c} \eta =UX_{\mathrm{ext}}, \end{array}$$

and we get μ=1,⋯,P constraint equations,

[5] $$\begin{array}{c} Z_{i,\mu }=\sum_{j=1}^{N_{y}}W_{i,j}\Phi \left(\eta_{j,\mu }+B_{j,\mu }\right). \end{array}$$

In these η-coordinates, the first P coordinates fully determine the input current UX. The last $N_{x}-P$ coordinates are for weights in the kernel of X, so they do not affect the input currents for the P conditions. Nevertheless, the synaptic weights depend on all columns of $X_{\mathrm{ext}}$ through

[6] $$\begin{array}{c} U=\eta X_{\mathrm{ext}}^{-1}. \end{array}$$

Note that this basis transformation acts independently on the incoming weights to each postsynaptic neuron. In order to change weights in the unconstrained dimensions, we make changes in η for all input conditions μ>P according to $\delta \eta_{i,\mu }^{U}∼N\left(0,\frac{\sigma }{\sigma_{s,\mu }}\right)$ (Fig. 3*D*), where σ sets the scale of the weight fluctuations, and $\sigma_{s,\mu }=\sqrt{\sum_{j=1}^{N_{x}}{\left(X_{\mathrm{ext}}^{-1}\right)}_{\mu ,j}^{2}}$ is chosen to generate equal variance weight fluctuations.

The second kind of flexibility arises due to the clipped-threshold-linear activation function, as changes in input currents do not affect activity if the input currents remains below the activity threshold or above the saturation threshold (Fig. 3*E*). Since these conditions lead to inequality constraints that restrict the solution space to part of a dimension, we refer to these as semiconstrained dimensions (32, 33). Importantly, which dimensions are semiconstrained can vary over time, as semiconstrained dimensions obtain new constraints when inactive or saturated neurons become engaged. Thus, diffusion in semiconstrained dimensions interacts with drift dimensions.

To coordinate changes in semiconstrained dimensions with representational drift, we divide neurons into sets of engaged ($E^{\mu }$), inactive ($I^{\mu }$), and saturated ($S^{\mu }$) neurons for each input condition μ≤P. These sets depend on time, but we will use elaborated notations like $E^{\mu ,t}$ only when it is important to emphasize this time dependence. The distinction between these sets is based on where the input current lies in relation to the thresholds, with neurons below the activity threshold (0) being inactive, ones above the saturation threshold (α) being saturated, and ones in between thresholds being engaged.

Neurons transition between these sets when their input current exactly corresponds to a threshold. For instance, for each neuron in the inactive set, we change its η-coordinates such that $\eta_{i,\mu }+\delta \eta_{i,\mu }^{I}+b_{i}\leq 0$, where $i\in I^{\mu }$. However, when a random change, $\delta \eta_{i,\mu }^{I}∼N\left(0,\frac{\sigma }{\sigma_{s,\mu }}\right)$, would have caused neuron i to cross its activity threshold at 0, we set the new $\eta_{i,\mu }$ to $-b_{i}$ and shift neuron i to engaged set (Fig. 3*F*). Similarly for saturated neurons, we ensure that $\eta_{i,\mu }+\delta \eta_{i,\mu }^{S}+b_{i}\geq \alpha$, where $i\in S^{\mu }$, and when the input current would have crossed the saturation threshold at α, we set the new $\eta_{i,\mu }$ to $\alpha -b_{i}$ and shift i to engaged set. These transitions between semiconstrained and engaged dimensions play a critical role in changing the sparsity of drift-explored solutions.

Diffusion in the drift dimensions typically requires concerted changes between neurons. By definition, this entails changes in representation that are orthogonal to the readouts. This implies that drift must be in the kernel of W. However, since small weight changes do not affect the activity of inactive and saturated neurons, drift can only change the activity of engaged neurons. Therefore, drift must locally occur in the kernel of the engaged submatrix, $W^{\mu }$, which only contains the columns of W corresponding to engaged neurons (Fig. 3*G*, *Left*). Since there are $N_{z}$ readout dimensions and $E^{\mu }=\left|E^{\mu }\right|$ engaged neurons for input μ, drift changes are within the $\left(E^{\mu }-N_{z}\right)$-dimensional kernel of $W^{\mu }$. As the engaged set for each input condition changes during drift, the engaged submatrix, the dimensionality of drift, and the drift dimensions change with time. We will use the explicit notation $E^{\mu ,t}$ when useful.

We sample drift changes as $\delta \eta_{i,\mu }^{E}=\sum_{j=1}^{E^{\mu }-N_{z}}\delta \eta_{i,\mu }^{E,j}k_{i^{\prime }\left(i\right)}^{j},$ where $\delta \eta_{i,\mu }^{E,j}∼N\left(0,\frac{\sigma }{\sigma_{s,\mu }}\right)$, $k^{1},\cdots ,k^{E^{\mu }-N_{z}}$ represent orthonormal basis vectors of $\ker W^{\mu }$, and $i^{\prime }:E^{\mu }\to \left\{1,\cdots ,E^{\mu }\right\}$ is a function that converts indices from $N_{y}$-dimensional vectors to $E^{\mu }$-dimensional vectors by replacing i with its position in $E^{\mu }$. For example, if $E^{\mu }=\left\{1,10,12\right\}$, then $i^{\prime }\left(1\right)=1$, $i^{\prime }\left(10\right)=2$, and $i^{\prime }\left(12\right)=3$. Due to the clipped-threshold-linear activation function, activity is bound between 0 and α, which imposes inequality constraints on representational drift akin to those for the changes in semiconstrained dimensions, $0\leq \eta_{i,\mu }+\delta \eta_{i,\mu }^{E}+b_{i}\leq \alpha$, where $i\in E^{\mu }$. However, if a neuron would have crossed one of the thresholds, we cannot simply set its η-coordinate to the threshold, since that change would no longer be in the kernel of $W^{\mu }$. Instead, we rescale the magnitude of the change so that the neuron sits exactly at threshold (Fig. 3*H*) (*Materials and Methods*). When more than one neuron would have crossed threshold, the rescaled change is the maximal that ensures that no neurons cross threshold. In either case, the neuron that reaches threshold is shifted from the engaged set to the inactive or saturated set depending on which threshold was reached. For instance, in Fig. 3*G* one neuron reaches the 0 threshold and becomes inactive. At the next time point, there is one fewer engaged neuron, and drift is limited to a lower-dimensional kernel.

By combining $\delta \eta^{U}$, $\delta \eta^{I}$, $\delta \eta^{S}$, and $\delta \eta^{E}$, we get δη as

[7] $$\begin{array}{c} \delta \eta_{i,\mu }=\left\{\begin{array}{c} \delta \eta_{i,\mu }^{U}\text{, if}\;\mu >P\\ \delta \eta_{i,\mu }^{I}|\;\eta_{i,\mu }+\delta \eta_{i,\mu }^{I}+b_{i}\leq 0\text{, if}\;i\in I^{\mu },\mu \leq P\\ \delta \eta_{i,\mu }^{S}|\;\eta_{i,\mu }+\delta \eta_{i,\mu }^{S}+b_{i}\geq \alpha \text{, if}\;i\in S^{\mu },\mu \leq P\\ \delta \eta_{i,\mu }^{E}|\;0\leq \eta_{i,\mu }+\delta \eta_{i,\mu }^{E}+b_{i}\leq \alpha \text{, if}\;i\in E^{\mu },\mu \leq P \end{array}\right.. \end{array}$$

We compute the new η-coordinates as η+δη and transform back to the physical coordinates using Eq. **6**. This method implements diffusive changes in η-coordinates, but the resulting δU is approximately normal with mean 0 and SD σ in physical coordinates (*SI Appendix*, Fig. S1).

### Allocation and Learning Without Interference.

**Fig. 4.**
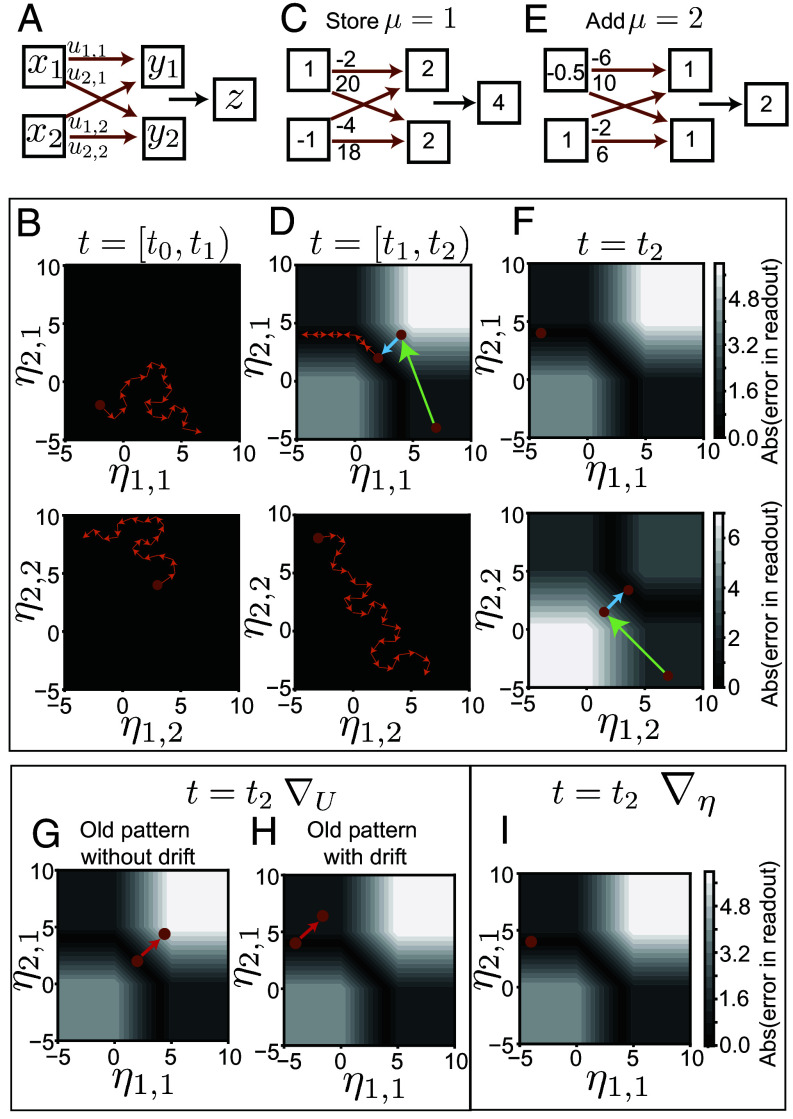
Allocation and continual learning. (*A*) Network architecture for a toy example, with two inputs, a representation layer with two neurons, and a single readout with fixed readout weights w=(1,1). (*B*, *D*, and *F*) The synaptic weights are visualized using η-coordinates based on the inputs that will be added to the network. Coordinates correspond to the first pattern $\eta_{\mu =1}$ (*Top*) and the second pattern $\eta_{\mu =2}$ (*Bottom*). (*B*) From time $\left[t_{0},t_{1}\right)$, no memories are added, and η (brown point) diffuses freely through drift (orange arrows). (*C* and *D*) At $t=t_{1}$ a new input–readout mapping from (1,−1) to 4 is to be stored (μ=1). The contour plots indicate the error surface in $\eta_{\mu =1}$ (*Top*). The green arrow denotes the allocation of $\eta_{\mu =1}$ to a dense representation (4,4), while the blue arrow shows gradient descent in $\eta_{\mu =1}$ to reach the solution space at (2,2). $\eta_{\mu =2}$ remains unchanged during allocation and learning (*Bottom*). Learned weights shown in *C*. Between $t_{1}$ and $t_{2}$ drift continues, with exploration constrained to the solution space in $\eta_{\mu =1}$ and free in $\eta_{\mu =2}$. (*E* and *F*) At $t=t_{2}$, a new input–readout mapping from (−0.5,1) to 2 is to be added (μ=2), generating an additional error surface (*Bottom*) on $\eta_{\mu =2}$. Allocation (green arrow) followed by gradient descent in η-coordinates (blue arrow) lead to the learned weights shown. (*G* and *H*) Gradient descent in U to learn μ=2, error contours with respect to μ=1. (*G*) Brown point indicates the coordinates due to learning of μ=1. The red arrow denotes the change in $\eta_{\mu =1}$ due to the addition of new mapping μ=2, resulting in large error for μ=1 mapping. (*H*) Brown point shows the coordinates after learning and drift (sparser solution). The red arrow shows change in $\eta_{\mu =1}$ due to new learning, resulting in small readout error for μ=1 because the postdrift solution is robust. (*I*) Gradient descent in η-coordinates to learn μ=2. Error contours with respect to μ=1, showing no change in performance for the previously learned memory.

Beyond defining the solution space, η-coordinates also enable targeted allocation and learning without interference. To understand these processes, consider a toy example with a two-neuron representation $\left(y_{1,\mu },y_{2,\mu }\right)$ that receives input from $\left(x_{1,\mu },x_{2,\mu }\right)$ via synaptic weights U and needs to generate 1-dimensional readouts $z_{\mu }$ for two sequentially learned mappings (μ=1,2) (Fig. 4*A*). We use readout weights w=(1,1), no bias (b=(0,0), and a clipped-threshold-linear activation function Φ (Eq. **2**) with activity and saturation thresholds at 0 and 5, respectively. Instead of directly visualizing the four-dimensional weight matrix U, we will use the basis transformed η-coordinates defined as

[8] $$\begin{array}{c} \left(\begin{array}{cc} \eta_{1,1} & \eta_{1,2}\\ \eta_{2,1} & \eta_{2,2} \end{array}\right)=\left(\begin{array}{cc} u_{1,1} & u_{1,2}\\ u_{2,1} & u_{2,2} \end{array}\right)\left(\begin{array}{cc} x_{1,1} & x_{1,2}\\ x_{2,1} & x_{2,2} \end{array}\right), \end{array}$$

where $\eta_{\mu =1}=\left(\eta_{1,1},\eta_{2,1}\right)$ and $\eta_{\mu =2}=\left(\eta_{1,2},\eta_{2,2}\right)$ are weight combinations relevant for each of the two input conditions, $x_{\mu =1}=\left(x_{1,1},x_{2,1}\right)$ and $x_{\mu =2}=\left(x_{1,2},x_{2,2}\right)$, respectively.

η-coordinates enable targeted allocation facilitating learning. Before learning, η freely explores via drift between time $t_{0}$ and $t_{1}$ (Fig. 4*B*). At $t=t_{1}$, when the system needs to learn a mapping from $x_{\mu =1}$ to $z_{\mu =1}$ (Fig. 4*C*), an error surface is introduced. The η-coordinates isolate the input-relevant weight combinations, so adding the first memory imposes an error surface on $\eta_{\mu =1}$ leaving $\eta_{\mu =2}$ with no constrains (Fig. 4*D*). Due to drift, the system is likely to start in a local optimum with both neurons disengaged for μ=1. Here, errors do not change locally, so it is unclear how to adjust weights for learning. To resolve this, we allocate the relevant weight combination, $\eta_{\mu =1}$, to random values centered in the engaged regime, leaving the weight combinations relevant to other inputs ($\eta_{\mu =2}$) unaffected (Fig. 4*D*). This moves the system into a densely engaged, gradient-rich, learnable regime. However, learning follows gradients to a nearby solution that is also densely engaged and thus nonrobust.

Drift subsequently makes the learned memories robust. Before adding memory μ=2, we allow the system to drift (Fig. 4*D*). It freely explores $\eta_{\mu =2}$ and diffuses in the $\eta_{\mu =1}$ solution space, often finding a sparsely engaged, robust solution.

At $t=t_{2}$, the system aims to add a new mapping μ=2 (Fig. 4*E*). This creates an error surface in $\eta_{\mu =2}$, while the error surface in $\eta_{\mu =1}$ remains unaffected because only $\eta_{\mu =2}$ includes weight combinations relevant to μ=2 (Fig. 4*F*). Again, we find that the configuration after drift generates representations with both disengaged neurons for μ=2, trapping it in a local optimum. As before, we can enable learning by allocating $\eta_{\mu =2}$ to a densely engaged learnable regime.

Drift helps reduce interference due to new learning. Learning is often modeled as gradient descent on the error function. The squared readout error for μ=2 is ${\left(J_{2}\right)}^{2}={\left(\sum_{i}w_{i}\Phi \left(\sum_{j}u_{i,j}x_{j,2}\right)-z_{2}\right)}^{2}$. To minimize the error, we update the weights in the direction opposite to the gradient,

[9] $$\begin{array}{c} \Delta u_{i,j}=-\lambda \left(\frac{\partial {\left(J_{2}\right)}^{2}}{\partial u_{i,j}}\right)\text{, where}\;\frac{\partial {\left(J_{2}\right)}^{2}}{\partial u_{i,j}}=2J_{2}w_{i}\Phi_{|\eta_{i,2}}^{\prime }x_{j,2}, \end{array}$$

$\frac{\partial J_{2}}{\partial u_{i,j}}$ is the gradient, λ is the learning rate, and $\Phi_{|\eta_{i,2}}^{\prime }$ is the derivative of Φ evaluated at $\eta_{i,2}$. Updating weights based solely on ${\left(J_{2}\right)}^{2}$, the error for μ=2, is likely to disrupt weight combinations relevant to previously stored memory (μ=1). This exemplifies the continual learning problem. The disruption severity depends on the robustness of stored memories. Densely engaged representations are less robust, thus more disrupted (Fig. 4*G*). While drifted memories are sparsely engaged, thus robust to weight updates (Fig. 4*H*).

Interference due to new learning can be completely eliminated using η-coordinates. In these coordinates, weight combinations relevant to each input condition are naturally segregated, so a new memory μ imposes its error surface only on its corresponding $\eta_{\mu }$. Thus, learning using gradient descent on the synaptic weights in η-coordinates,

[10] $$\begin{array}{c} \Delta \eta_{i,2}=-\lambda \left(\frac{\partial {\left(J_{2}\right)}^{2}}{\partial \eta_{i,2}}\right)\text{, where}\;\frac{\partial J_{2}}{\partial \eta_{i,2}}=2J_{2}w_{i}\Phi_{\eta_{i,2}}^{\prime }, \end{array}$$

modifies the weight combinations relevant to the new memory while preserving those associated with stored memories, $\Delta \eta_{i,1}=0$ (Fig. 4*I*). When the number of input neurons exceeds the number of memories ($N_{x}>P$), η-based learning circumvents the continual learning problem, enabling sequential addition of memories without the need for batch learning. However, the η-coordinates of the weights are defined using a basis transformation matrix $X_{\mathrm{ext}}$ that incorporates all inputs, leaving the biological implementation unclear (*Discussion*).

### Unbiased Exploration of Weight Solution Space Favors Sparsely Engaged, Robust Solutions.

**Fig. 5.**
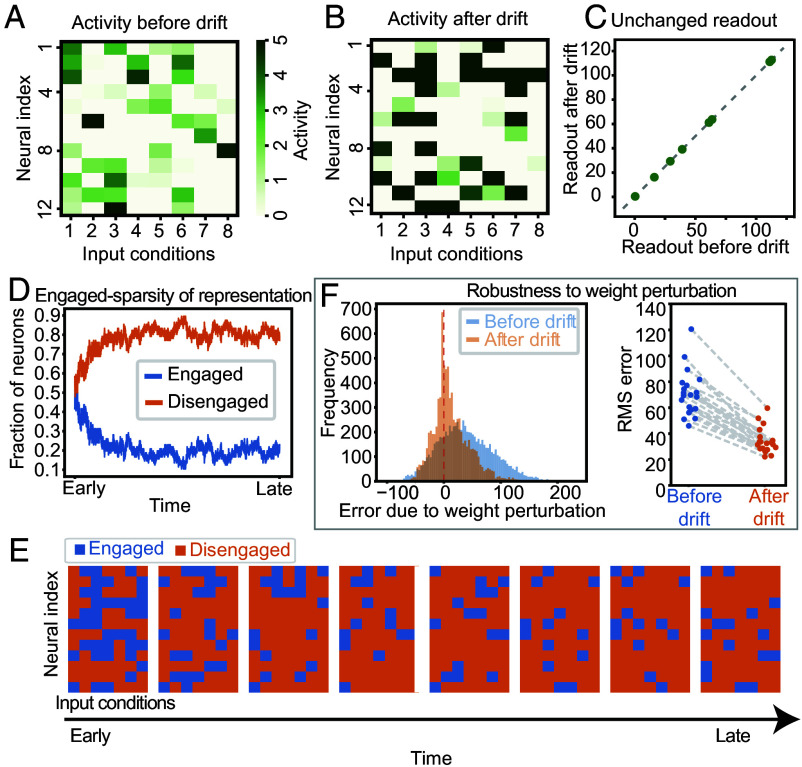
Representational drift leads to sparsely engaged representations that are robust to weight perturbations. (*A*) Activity of 12 neurons at 8 different input conditions found using gradient descent. (*B*) Neural activity after simulating drift as a diffusion in the solution space. (*C*) The readout of the representation before and after drift remains the same. (*D*) Fraction of engaged and disengaged (inactive and saturated) neurons over time during drift, showing an initial drop in the fraction of engaged neurons followed by stable maintenance. (*E*) Engaged and disengaged neurons for each input condition over time during drift. (*F*) Histogram of signed errors in readout due to U perturbations ∼N(0,1) (*Left*). RMS error due to weight perturbations before drift (blue) and after drift (orange) (*Right*).

We simulated representational drift and studied the properties of the resulting representations. As anticipated, the representations changed (Fig. 5 *A* and *B*) while the low-dimensional readouts remained stable (Fig. 5*C*). During initial drift, the number of engaged neurons decreased, leading to sparsely engaged representations (Fig. 5*D*). Throughout later drift, the fraction of engaged neurons remained stably low, although the specific neurons in the engaged set continued to change (Fig. 5*E*). Under weight perturbations, sparsely engaged solutions explored by drift yielded smaller readout errors than the learned solutions (Fig. 5*F*). Thus, uniform sampling of the synaptic weight solution space resulted in a biased sampling of sparsely engaged, noise-robust solutions.

### Representational Drift Increases Robustness at the Expense of Learnability.

**Fig. 6.**
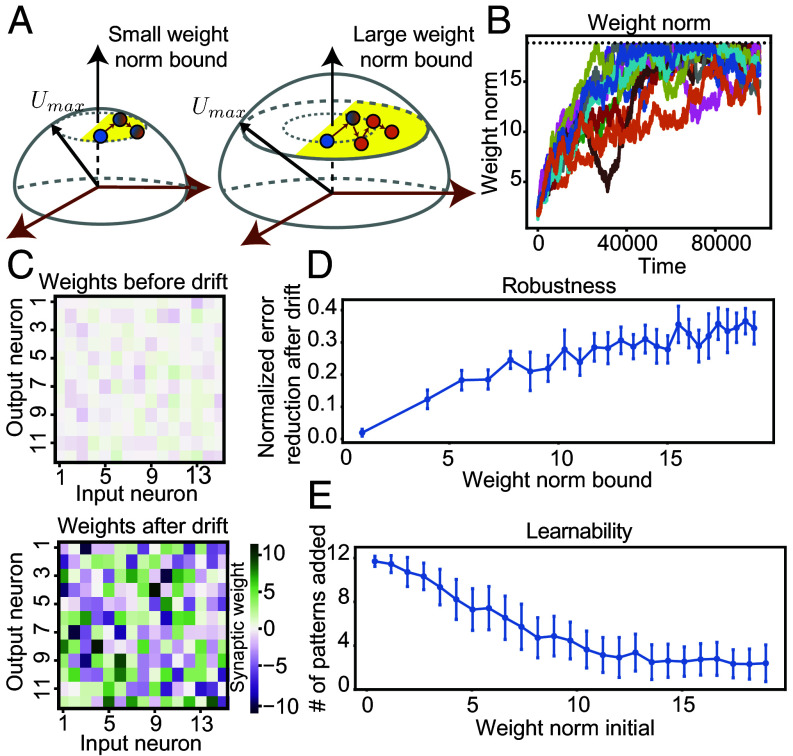
Representational drift increases robustness at the expense of learnability. (*A*) Schematic illustration of a small weight norm bound and a large weight norm bound, highlighting that densely engaged, nonrobust solutions (blue) are more prevalent for small weight norms, while sparsely engaged, robust solutions (orange) are more common for large weight norms. (*B*) Weight norms onto each postsynaptic neuron (different colors) over time during drift, showing an increase and plateau close to the enforced bound (black dotted line). (*C*) Small magnitude weights postlearning and large magnitude weights postdrift. (*D*) Robustness: difference between readout error due to weight perturbations before and after drift, scaled by the error before drift. Robustness increases with weight norm bound. (*E*) Learnability (number of input–readout mappings successfully learned) decreases with initial weight norms.

Representational drift caused an increase in weight magnitudes. To ensure finite weights during drift, we imposed a weight norm bound $U_{\max}$ for each postsynaptic neuron j, $\sum_{i=1}^{N_{x}}U_{j,i}^{2}\leq {\left(U_{\max}\right)}^{2}$ (Fig. 6*A*). In high-dimensional spheres, most of the volume is concentrated near the surface, meaning there are more weights with norms close to the imposed bound than smaller norms. Thus, drift entropically favored solutions with weight norms close to $U_{\max}$. Further, $U_{\max}$ greatly exceeded the weight norms of the initially learned solution, so during drift, weight norms increased to reach $U_{\max}$ and fluctuated close to it (Fig. 6*B*). Thus, synaptic weights after drift were substantially larger than those before (Fig. 6*C*).

Large weight solutions explored by drift enhanced robustness. Higher weight magnitudes generate larger input currents, causing many neurons to fall below the activity threshold or exceed the saturation threshold, leading to more disengaged neurons (*SI Appendix*, section 2B). The greater the number of disengaged neurons, the greater the robustness to weight perturbations (*SI Appendix*, section 2C and Fig. S6). As a result, the robustness benefit from drift increased with the weight norm bound (Fig. 6*D*).

Large weight solutions explored by drift hindered future learning. After drift, the system must continue to learn. However, large weights generated by drift produce large currents for new inputs, leading to sparsely engaged representations. This causes the gradients to vanish, preventing the system from learning new information. Consequently, the ability to learn decreased as initial weight magnitudes increased (Fig. 6*E*). This creates a trade-off between retaining robust memories and the capacity to learn new information, with larger weights favoring robustness but limiting learnability (Fig. 6 *D* and *E*).

### Combining Drift with Allocation Overcomes the Learnability–Robustness Trade-Off and Enables Continual Learning.

**Fig. 7.**
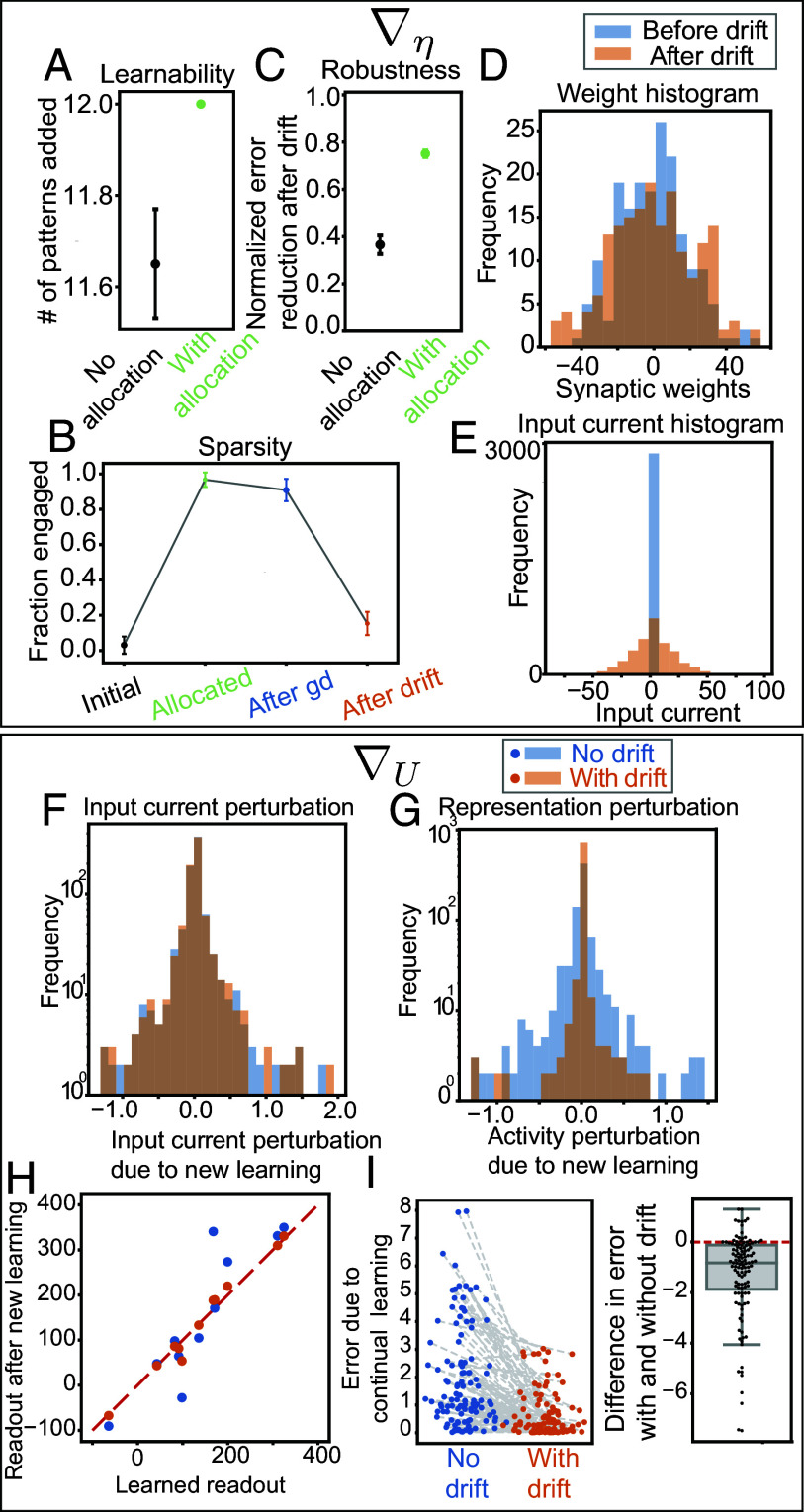
Allocation and drift overcome the learnability–maintainability trade-off and lessen the continual learning problem. (*A*) The mean and SE of the number of input–readout mappings that were successfully added to the network, with and without allocation. “No allocation” shows the best learnability case from (Fig. 6*E*), with weight norm = 1. (*B*) Fraction of engaged neurons at different stages: initially (before allocation), after allocation, after learning, and after drift. (*C*) Difference between error in readout caused by random weight perturbations before and after drift, scaled by the error before drift, with and without allocation. No allocation is the best robustness case from (Fig. 6*D*), with weight norm = 350. (*D*) Histogram of weights U before and after drift, showing weights remain comparable. (*E*) Input currents before and after drift, showing drift causes input currents to spread out. (*F*–*I*) Continual learning simulations performing gradient descent in U, without drift (blue) and with drift (orange) in between, to evaluate the effect of learning new pattern ν on previously stored patterns μ. (*F*) Histogram of perturbations to input currents $\eta_{\mu }$ due to new learning ν, showing same effect regardless of drift. (*G*) Histogram of changes to neural representation $Y_{\mu }$, indicating larger changes without drift compared to with drift. (*H*) For each pattern μ, readout upon learning μ vs. readout after learning new pattern ν, with $\mu <\nu \leq N_{x}$. (*I*) RMS error in readout for pattern μ after learning of newer pattern ν and the difference between readout errors with drift and without drift, highlighting reduced errors with drift compared to without drift.

Targeted allocation improved learnability despite large weight magnitudes. We initialized the network with large synaptic weights, U∼N(0,15), and incrementally added new memories μ=1,⋯,P. For each memory μ, we first allocated the relevant weight combinations $\eta_{\mu }∼N\left(2.5,0.1\right)$, near the center of the engaged regime, (0, 5). Next, we performed gradient descent learning in $\eta_{\mu }$, modifying only weight combinations relevant to the μth input. Before learning the next pattern, we allowed the system to drift while preserving readouts for all learned patterns 1,⋯,μ. Using this approach, we successfully incorporated $N_{x}$ memories, slightly surpassing the learnability achieved with small weight initializations (Fig. 7*A*). While small weight initializations avoid saturation, they are equally likely to generate both inactive and engaged neurons. In contrast, the allocation method centers input currents within the engaged regime, ensuring densely engaged initializations (Fig. 7*B*), enhancing gradient availability, and thus learnability.

Drift can sparsify and enhance robustness without increasing weight magnitudes. Starting from densely engaged initializations, gradient descent caused some neurons to disengage, but the learned solutions remained densely engaged (Fig. 7*B*) and thus nonrobust to weight perturbations. Drift improved robustness (Fig. 7*C*) while maintaining similar weight distributions (Fig. 7*D*). The allocation procedure made the representations more densely engaged than typical for the set weight norm bound. During drift, the system explored more typical solutions with larger input currents (Fig. 7*E*), resulting in sparsely engaged (Fig. 7*B*), robust solutions (Fig. 7*C*).

Drift improved robustness to continual learning. We incrementally added new memories μ=1,⋯,P by allocating $\eta_{\mu }$ and performing gradient descent on the synaptic weights U for the new memory μ. In one case, the system drifted between memory additions, while in another memories were added without drift. In both cases, new learning caused the same input current perturbations to previously stored memories (Fig. 7*F*). However, because drift found sparsely engaged representations, the same input current perturbations had less impact on the neural representations (Fig. 7*G*) and, consequently, the readouts (Fig. 7*H*), resulting in lower readout errors (Fig. 7*I*). Thus, by increasing robustness to weight perturbations, drift improves memory maintenance during new learning.

## Discussion

### The Purpose of Representational Drift.

Representational drift has been proposed to improve learning and memory. For instance, drift may help revise memories in light of new experiences (12, 39) or provide a baseline level of synaptic flux that helps overcome the exploration–exploitation dilemma in dynamic environments (15). Drift can prevent the system from getting stuck in local minima (11, 40) or improve generalization through drop-out regularization (26, 41). Other interesting proposals include probabilistic inference (16, 31), time stamping of memories (7), systems consolidation (39, 42), and memory decay to prevent catastrophic forgetting (12).

Our theory provides interesting insights on the role of drift in overcoming the plasticity-stability dilemma. Many previously proposed advantages arise from enhanced exploration of different memories in dynamic environments that demand memories to change in order to align with the current context. Our work shows that representational drift can also provide benefits when memories are stably maintained, as it can mitigate interference caused by noise and continual learning. This provides an intriguing perspective on how representational drift relates to continual learning. Many have suggested that representational drift is a byproduct of error-corrective mechanisms that bring the system back to the solution space following perturbations caused by noise and continual learning (11, 15, 41). Since we show that representational drift enhances robustness to noise and continual learning, this suggests that representational drift may fortify the system against the very processes that give rise to it. Our proposal that drift benefits continual learning through enhanced stability likewise offers a different perspective on the stability–plasticity dilemma. Traditionally, stability is considered crucial for retaining memories and plasticity essential for learning new information. Thus, the stability–plasticity dilemma arises from the competing demands to balance memory maintenance and new learning (43, 44). In contrast, our results challenge the notion of a direct competition between plasticity and stability. Instead, plasticity-induced drift may actually bolster stability.

### Representational Drift as Memory Consolidation.

In our model, drift shifts memories from less stable to more stable states, a role conceptually similar to memory consolidation. Various models of memory consolidation have suggested ways to stabilize memories after learning. For instance, Fusi et al. proposed metaplastic transitions into less plastic synaptic states (45), elastic weight consolidation protects task-relevant synapses during learning (46), and Roxin et al. transfer memories to less plastic systems via replay (47). Our framework differs from these prior models by proposing that memory stabilization may not always require stable synaptic states, as the sparse representations most frequently sampled through drift are inherently more stable than those initially found by learning.

The memory stability provided by drift could be compared to previous memory consolidation models through their robustness to ongoing learning. Using η-coordinates, we show how allocation, learning, and drift can be coordinated to fully eliminate the interference between memory and learning. However, this requires the intricate coordination of many separate synapses, and our current construction only applies in the special case where a single hidden-layer neural network stores fewer memories than it has input neurons. In contrast, other models support greater memory loads and less-restrictive architectures but do not entirely prevent interference. Importantly, these mechanisms are not mutually exclusive, and they may all contribute to the robustness demanded by biology.

### Disentangling Drift from Learning.

Our work demonstrates the power of mathematical modeling in disentangling representational drift from learning–two highly intertwined processes. For instance, if representational drift results from ongoing learning (11), then the same biological mechanisms underlie both, making it experimentally challenging to isolate them. Nevertheless, here we mathematically isolated drift by modeling it as weight space diffusion in readout-irrelevant dimensions, analytically identified by extending prior theoretical work on the geometry of neural network solution spaces (32, 33). With perfect task performance and drift-induced changes orthogonal to the required readouts, we isolated benefits stemming solely from random exploration of the solution space. Moreover, this abstraction allowed our model to remain agnostic to what drives drift, whether that be ongoing learning, stochastic synaptic changes (15, 48, 49), spiking noise (50), noisy gradients (51), or fluctuating neuronal excitability (52).

This separation revealed that several phenomena typically associated with learning can also result from representational drift of stably maintained memories (*SI Appendix*, section 3). For instance, representations tend to decorrelate over time (53–55), reducing interference and improving separability (55–58), and this has often been attributed to learning. However, our work suggests that drift can also help orthogonalize memories. In high-dimensional spaces, two randomly chosen vectors are close to orthogonal (59). Therefore, even if representations for two stimuli are initially correlated, perhaps due to temporal proximity of encoding (60, 61) or input similarity, representational drift decorrelates them over time (*SI Appendix*, section 3C and Fig. S9). Similarly, our work suggests that representational sparsification previously associated with learning may arise from drift alone due to the geometry of the solution space (*SI Appendix*, section 3B and Fig. S8). For instance, a closely related study by Ratzon et al. observed sparsification of neural representations driven by implicit regularization during learning, followed by stable sparsity during a subsequent drift-like learning phase involving random diffusion within a low-loss, high-regularization manifold (41). In their model, sparsification emerged because noisy learning implicitly favored robust solutions. In contrast, sparsification arises in our framework from undirected random drift that naturally explores the more prevalent sparse solutions, not from any preference for robust solutions. Finally, we note that our model generates a decrease in representational change with familiarization (*SI Appendix*, section 3A and Fig. S7). This effect would intuitively suggest a reduction in learning-related updates over time, but we show that random diffusion alone can produce a similar pattern, with larger representational changes early on and slower drift at later stages.

### Biologically Plausible Mechanisms for Allocation.

We considered a theoretically convenient yet abstract model of allocation. Here, we relate the core elements of the model to candidate underlying mechanisms.

First, our model performs allocation prior to discrete learning events, but the triggers for allocation might be more complex in biology. Our results suggest that allocation can help encode new input–output mappings, so novelty detection could trigger allocation (62), especially when there are new sensory-behavior associations (63). More generally, allocation could be driven by whatever mechanisms the brain uses to prioritize information for memory encoding (64, 65). Since we proposed allocation to escape local optima, allocation may be triggered by failed learning events. However, it is unclear whether the brain detects such failures.

Second, we implement allocation by resetting synaptic weights to values that provide gradients, but this is only one of several mechanisms that biology could use. Directly instantiating our memory allocation model would require input-dependent mechanisms that coordinate synaptic weight changes across many synapses. This is not inconceivable, as the required shifts can be subtle, and homeostatic plasticity can rescale many synapses onto a postsynaptic cell (66) and selectively modify activated synapses (67–69). Nevertheless, a more attractive alternative might involve transiently altering neuronal excitability, a mechanism widely associated with allocation in biology (70, 71). In our framework, changes in excitability could correspond to changes in baseline or thresholds, thereby increasing neuronal engagement and providing gradients for learning. These changes could be made transient by homeostatically restoring excitability in concert with compensatory weight changes to preserve relevant readouts.

Third, our model used the geometry of the solution space to allocate and learn without disrupting stored memories, but this is not a literal proposal for how biology addresses continual learning. Allocation and learning can completely avoid interference by modifying synaptic weights along the intended η-coordinates. However, in biology, plasticity must occur in the coordinate system of individual synapses, and determining the required weight changes in η-coordinates requires knowing all past, current, and future input patterns (Eq. **6**). There is some evidence that memory representations are fixed prior to memory encoding (72), so biology may learn with predetermined representations whose statistics make it easy to learn in η-coordinates. For instance, if the input patterns are all orthogonal to each other, then allocating and learning in η-coordinates merely requires synaptic plasticity based on new input patterns. While this is unlikely for typical learning, it illustrates what a brain must somehow accomplish to achieve its impressive continual learning capabilities (73). In less restrictive conditions, allocation could occur by transiently changing the neuronal excitability, as discussed above. Nevertheless, the learning required to store new mappings and restore the original excitability would typically degrade older memories.

### Toward Empirically Accurate Models of Representational Drift.

The solution space framework developed here can be extended to more biologically realistic scenarios, offering deeper insights into solutions explored by drift. Prior studies have suggested that drift in certain brain regions cannot be explained by a fixed linear readout (19, 36). Interestingly, our simulations show that even when representations drift with a fixed linear readout, limited access to the neural population can make this difficult to detect (*SI Appendix*, section 3D and Fig. S10). Nevertheless, weights could change across multiple regions, so it is important to adapt our framework to incorporate multiple neural layers and plastic readout weights. Our preliminary analyses show that sparse solutions remain common when representations and readout weights drift independently (*SI Appendix*, section 1B and Fig. S2). Future work should account for coordinated changes. Finally, while this study modeled the readout as a linear projection, experimental evidence predominantly highlights nonlinear stable subspaces that persist despite drift (20, 23–25). Extending the framework to experimentally observed nonlinear readouts is a promising direction for future work.

Biologically realistic models of representational drift also demand that parameter values be chosen to mimic experimental results. For instance, in this study’s proof-of-concept models, we classified both inactive and saturated neurons as disengaged because they both appeared frequently in the solution space and enhanced robustness to perturbations that posed challenges for gradient-based learning. Yet inactive neurons are far more prevalent than saturated ones in biology (74). Our model can mimic this outcome through parameter modifications (*SI Appendix*, section 3C and Fig. S8). Moreover, we find that drift in these networks produces more biologically realistic single-cell dynamics, including intermittent firing and shifts in activity fields (36, 75). These sparsely active representations retain the robustness benefits highlighted for sparsely engaged ones and also offer additional advantages, like energy efficiency (76–78) and robustness to readout changes.

### Further Extensions to the Theoretical Framework.

Future work should relax theoretical assumptions made for mathematical convenience, which may not be biologically correct. First, we assumed that the number of memories is smaller than the number of input neurons ($P\leq N_{x}$), which enabled us to find a basis that separates out relevant weight combinations (32, 33). The existence of representational drift suggests that the brain may be highly redundant and operate below its memory capacity (11, 79), but this capacity can exceed the number of input neurons. Therefore, extending our framework to define the solution space manifold near the network’s full capacity would be valuable. Second, drift may not be restricted to exact solutions but may tolerate small errors in the readouts (15, 41, 51), so it is important to generalize the framework to accommodate nonzero error solution spaces (32).

Extensions to the modeling framework could reveal more robust higher-dimensional solution spaces. In this work, we observed that the solution space dimensionality was the same for densely engaged and sparsely engaged solutions (*SI Appendix*, section 2). However, if certain regions of the solution space were higher-dimensional, then this would provide a plethora of even more robust solutions. We observed such high-dimensional solution spaces in toy examples for specific readout values and activity thresholds (*SI Appendix*, section 2A and Fig. S4), but they did not emerge in drift simulations (*SI Appendix*, sections 2B and 2C). Such solutions might be absent in the simulated examples and may become prevalent in models with dynamic neuronal excitability, where thresholds can be tuned (80, 81), or in models allowing nonzero readout errors (35). It is also possible that higher-dimensional solution spaces exist but are hard to diffuse into. Future work calculating the volume and dimensionality of solution spaces may help determine whether drift can enhance robustness using higher-dimensional solution spaces.

### Model Predictions and Relation to Experimental Findings.

Our model reproduces experimental findings regarding changes in sparsity of representations. In our model, we find changes in engaged sparsity, or, in a more biologically relevant context (*SI Appendix*, section 3B and Fig. S8), activity sparsity, suggesting that representations become dense to facilitate learning, sparsify during early drift, and maintain this sparsity during later drift. This aligns with findings in the CA1 hippocampus, a region known to have extensive drift (6, 8). In this region, activity in a novel environment increases through recruitment of additional cells, which is followed by sparsification with familiarization (82–85). Similarly, in the somatosensory cortex, another region exhibiting drift, neurons transiently increase activity during training before sparsifying (21, 63). While sparsification has been linked to learning (41, 85) or behavioral changes (20), our model suggests that it can arise due to solution space exploration. In contrast, regions with limited drift like CA3 do not exhibit sparsity changes with familiarization (5, 86). Further supporting our findings, the fraction of active neurons remains stable during later drift (5, 7).

A key insight of our model is that the rate of representational drift can vary even when synaptic dynamics remain constant. In our framework, synaptic changes onto disengaged neurons do not affect representations unless thresholds are crossed, so sparsely engaged representations drift less than densely engaged ones. This provides a potential explanation for why natural movies in V1, which elicit dense representations, exhibit greater drift, whereas sparser representations evoked by gratings are more stable (87, 88). In our model, early in drift, representations are densely engaged, so they drift more. Over time, as representations sparsify, drift slows (*SI Appendix*, Fig. S7). Moreover, as disengaged neurons explore further into semiconstrained dimensions, they become less likely to re-engage, further reducing drift. This decline in representational change aligns with experimental findings (22, 75, 89, 90) and computational studies (41). Though learning likely contributes to decreasing representational turnover, our model shows that drift alone can account for such decline.

In addition to reproducing experimental observations, our model makes predictions about subthreshold input currents. In our framework, representational drift leads to sparsely engaged representations because there are many input current configurations that yield sparse representations. Thus, our model predicts that drift leads to large and variable subthreshold input currents onto disengaged neurons. For inactive neurons, we predict highly variable input currents well below the activity threshold (*SI Appendix*, Fig. S8). We consider this our key prediction because it challenges prevailing theories and remains to be tested. Unlike continual learning models that achieve robustness by stabilizing synapses, our model stabilizes representations by exploring semiconstrained dimensions of subthreshold input currents, making this prediction distinctive to the model. Moreover, it stands in contrast to balanced network theory, which posits near-threshold membrane potentials maintained through balanced excitation and inhibition (91). While excitation–inhibition balance is observed under anesthesia, in awake states relevant to memory, inhibition may dominate, resulting in membrane potentials far below threshold (92–96). Crucially, variability in subthreshold membrane potentials remains unexplored in brain regions showing drift. Our model suggests that following learning, the subthreshold input currents onto inactive neurons should lie close to the activity threshold. With drift, these input currents should diffuse and reach far below threshold if those neurons remain inactive. Studying the variability and evolution of subthreshold membrane potentials will help test these predictions.

## Materials and Methods

We established a task framework and neural network architecture to study representational drift. Elements of the input matrix X, comprising the activity of $N_{x}=15$ input neurons across P=8 input conditions, were drawn with mean 0 and SD 1 as $X_{i,\mu }∼N\left(0,1\right)$. The representation layer consisted of $N_{y}=12$ neurons and its activity Y was used to generate an $N_{z}=1$-dimensional readout through a fixed readout weight matrix $W_{i,j}∼N\left(0,10\right)$. We used a clipped-threshold-linear activation function with an activity threshold of 0 and a saturation threshold of α=5, unless specified otherwise. The desired readout was specified by a teacher network, which processes the input through a weight matrix T, applies the same clipped-threshold-linear activation function, and performs a one-dimensional linear projection via the same weights W. Thus, the desired readouts are given by WΦ(TX+B), while the readout generated by the network is given by WΦ(UX+B), where the bias term, B, was set to a zero matrix unless stated otherwise.

To simulate drift as an exploration of the solution space, we first needed to reach the solution space through learning. The weights U were initialized as U∼N(0,0.1) unless specified otherwise. We allocated the weight combinations relevant to the P input conditions within the center of the engaged regime by setting $\eta_{\mu }∼N\left(\frac{\alpha }{2},0.1\right)$, where 0≤μ<P. Subsequently, we updated the corresponding weights U using gradient descent (Adam optimizer in PyTorch, with a learning rate of 0.001) to minimize the mean squared error (MSE) between the network readouts and the readouts generated by the teacher network across P input conditions. The readout weights W and the bias term B were kept fixed unless specified otherwise. When adding the $\mu^{\mathrm{th}}$ memory in the continual learning setting with gradient descent directly in U (Fig. 7*F*–*I*), we allocated $\eta_{\mu }$ and updated the corresponding weights U to minimize the loss only for the new memory μ using Eq. **9**. Similarly, for continual learning with gradient descent in η-coordinates (Fig. 7*A*–*E*), we allocated $\eta_{\mu }$ and then updated it to reduce the loss for the $\mu^{\mathrm{th}}$ input condition using Eq. **10**.

We would like to acknowledge the use of OpenAI’s ChatGPT for assisting with language and grammar refinement and Adobe Illustrator’s generative AI for help creating Fig. 1*A*.

## Supplementary Material

Appendix 01 (PDF)

## Data Availability

The code used in this study is available on GitHub (97). All other data are included in the article and/or *SI Appendix*.
