# Supplementary material for: Stability through plasticity: Finding robust memories through representational drift
Source: Proc Natl Acad Sci U S A. 2025 Nov 7;122(45):e2500077122. doi: 10.1073/pnas.2500077122 (PMC12625983; doi:10.1073/pnas.2500077122)
Supplement: Supplementary file 1 — Appendix 01 (PDF) [file pnas.2500077122.sapp.pdf]

# Supporting Information Text

## 1. Model variants

**A. Generalizing to other non-linear continuous activation functions.** In this section, we generalize the proposed solution space manifold setup to accommodate a broader class of non-linear activation functions. Consider a network where the representation-layer activity is given by $Y_{i,\mu} = \Psi\left(\sum_j U_{i,j} X_{j,\mu}\right)$, where $Y_{i,\mu}$ is the activity of the $i^{th}$ neuron under input condition $\mu$, $U_{i,j}$ is the synaptic weight from input neuron $j$ to representation-layer neuron $i$, $X_{j,\mu}$ is the input activity of neuron $j$ under input condition $\mu$, and $\Psi$ represents a continuous activation function defined as

$$\Psi(v) = \begin{cases} f(a), v \leq a \\ f(v), a < v < b \\ f(b), v \geq b \end{cases}, \quad [1]$$

where $f(v)$ is a strictly increasing function, ie. if $a \leq v_1 < v_2 \leq b$, then $f(v_1) < f(v_2)$. We define another function $\Omega$

$$\Omega(\omega) = \begin{cases} 0, \omega = f(a) \\ f^{-1}(\omega) - a, f(a) < \omega < f(b) \\ b - a, \omega = f(b) \end{cases}. \quad [2]$$

Then

$$\Omega\left(\Psi(v+a)\right) = \begin{cases} 0, v \leq 0 \\ f^{-1}(f(v+a)) - a, 0 < v < b - a \\ b - a, v \geq (b-a) \end{cases} = \begin{cases} 0, v \leq 0 \\ v, 0 < v < b - a \\ b - a, v \geq (b-a) \end{cases} = \Phi_{0,b-a}(v), \quad [3]$$

where $\Phi_{0,b-a}(v)$ is a clipped-threshold linear activation function with an activity threshold of 0 and a saturation threshold of $b - a$. If we define $\widetilde{Y}_{i,\mu} = \Omega(Y_{i,\mu})$, then the original network with $\Psi$ activation function becomes equivalent to a network with clipped-threshold linear activation function $\Phi_{0,b-a}$ with an activation threshold of 0, a saturation threshold of $b - a$ and a baseline activity or bias of $-a$

$$\widetilde{Y}_{i,\mu} = \Phi_{0,b-a}\left(\sum_j U_{i,j} X_{j,\mu} - a\right). \quad [4]$$

**B. Generalizing to dynamic readout weights.** In this paper, we modeled representational drift by generating different representations that satisfy a particular input-output mapping with fixed abstract readout weights. Here, we extend our approach to a scenario where both the input synaptic weights $U$ and the abstract readout weights $W$ are subject to instability (Fig. S2A). We do so by alternating between two steps: (1) keeping $W$ fixed and identifying changes in $U$ that preserve the downstream readout, and (2) keeping $U$ (and subsequently $Y$) fixed and determining changes in $W$ that maintain a fixed readout (specifically, changes in the null space of $Y$).

Previously, instead of making changes directly in $U$, we made changes in a transformed version of $U$ ie. $\eta$. Similarly, we make changes in a basis transformed version of $W$, ie. $\zeta$ We consider the case where the number of neurons in the hidden layer is greater than the number of stimuli stored ie. $N_y > P$,

$$\zeta_{j,\mu} = \sum_{k=1}^{N_y} W_{j,k} (Y_{ext})_{k\mu}, \quad [5]$$

where $Y_{ext}$ is computed from $Y$ by stacking $N_y - P$ columns in the null space of $Y$ to it. Then we make changes $\delta\zeta$ as follows:

$$\delta\zeta_{j,\mu>P} = \mathcal{N}\left(0, \frac{\sigma}{\sigma_{s,\mu}}\right) \text{ where } \sigma_{s,\mu} = \sqrt{\sum_{i=1}^{N_y} \left(Y_{ext}^{-1}\right)_{\mu,i}^2}. \quad [6]$$

Similar to the weight norm bound set to the hidden layer neurons, for each dimension of the readout $k$, we impose a bound on the readout weights such that

$$\sum_{j=1}^{N_y} W_{k,j}^2 \leq (W_{max_k})^2, \quad [7]$$

where $W_{max_k}$ is the maximum weight norm allowed onto the $k^{th}$ readout dimension. When a proposed $\delta\zeta$ causes the weights onto readout $k$ to exceed its limit $\sum_{j=1}^{N_y} W_{k,j}^2 > (W_{max_k})^2$, then we scale the unconstrained dimensions of $\delta\zeta$ such that the weight norm is set to the bound ie. $\sum_{j=1}^{N_y} W_{k,j}^2 = W_{max_k}$. Thus, we modified synaptic weights $U$ and readout weights $W$ (Fig. S2B-D), while preserving the relevant readouts $Z$ (Fig. S2E).

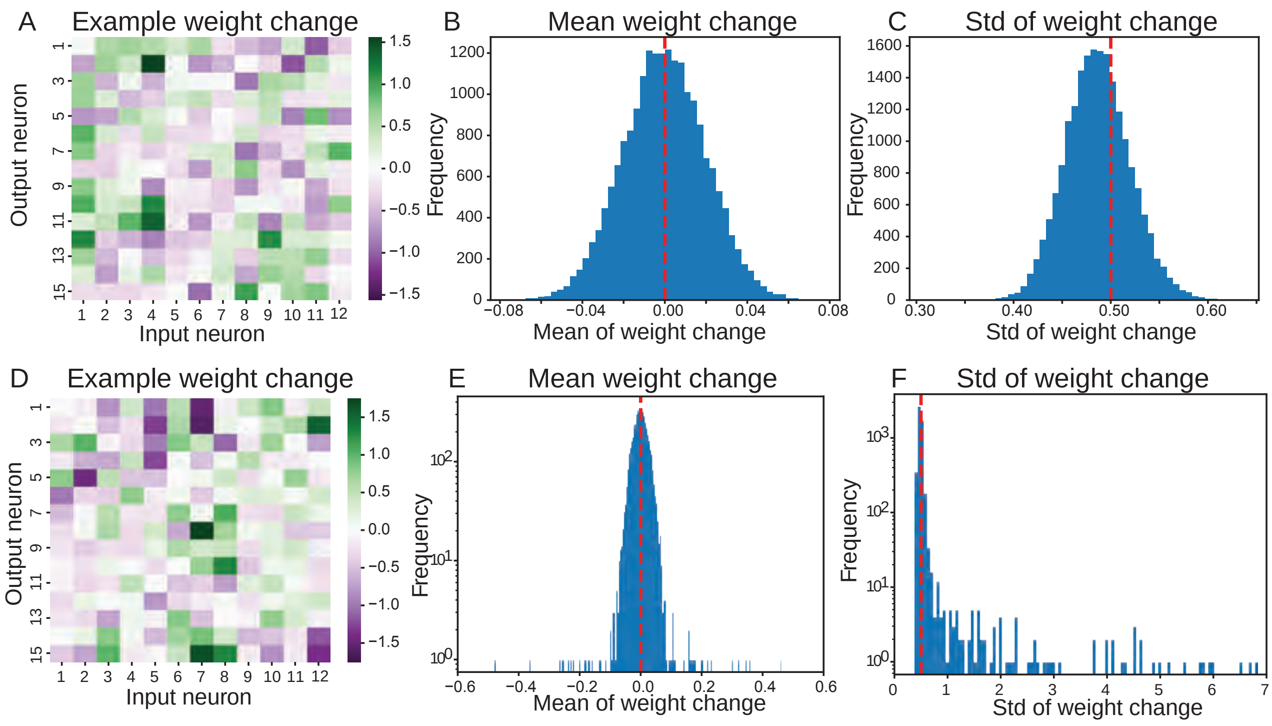


**Fig. S1.** Characterization of synaptic weight changes during drift. (A-C) Drift without any weight norm bound. (A) An example $dU$ resulting from uncorrelated $\eta$ change. (B) The distribution of mean weight changes, with red dotted line indicating the expected mean of 0 (C) The distribution of the standard deviation of weight changes, which is slightly smaller than the expected standard deviation ($\sigma = 0.5$, marked by the red dotted line) possibly due to rescaling of changes done to avoid threshold crossing. (D-F) Drift with weight norm bound set to its initial value for each neuron. (D) Example weight change $dU$. (E) Distribution of mean weight changes is still centered around 0. However, some weight changes exhibit means further away from 0 due to additional changes made to ensure weight norm constraint. (F) The distribution of the standard deviation of weight changes remains close to the expected $\sigma = 0.5$, although there are instances of larger weight changes, made to adhere to weight norm constraints.

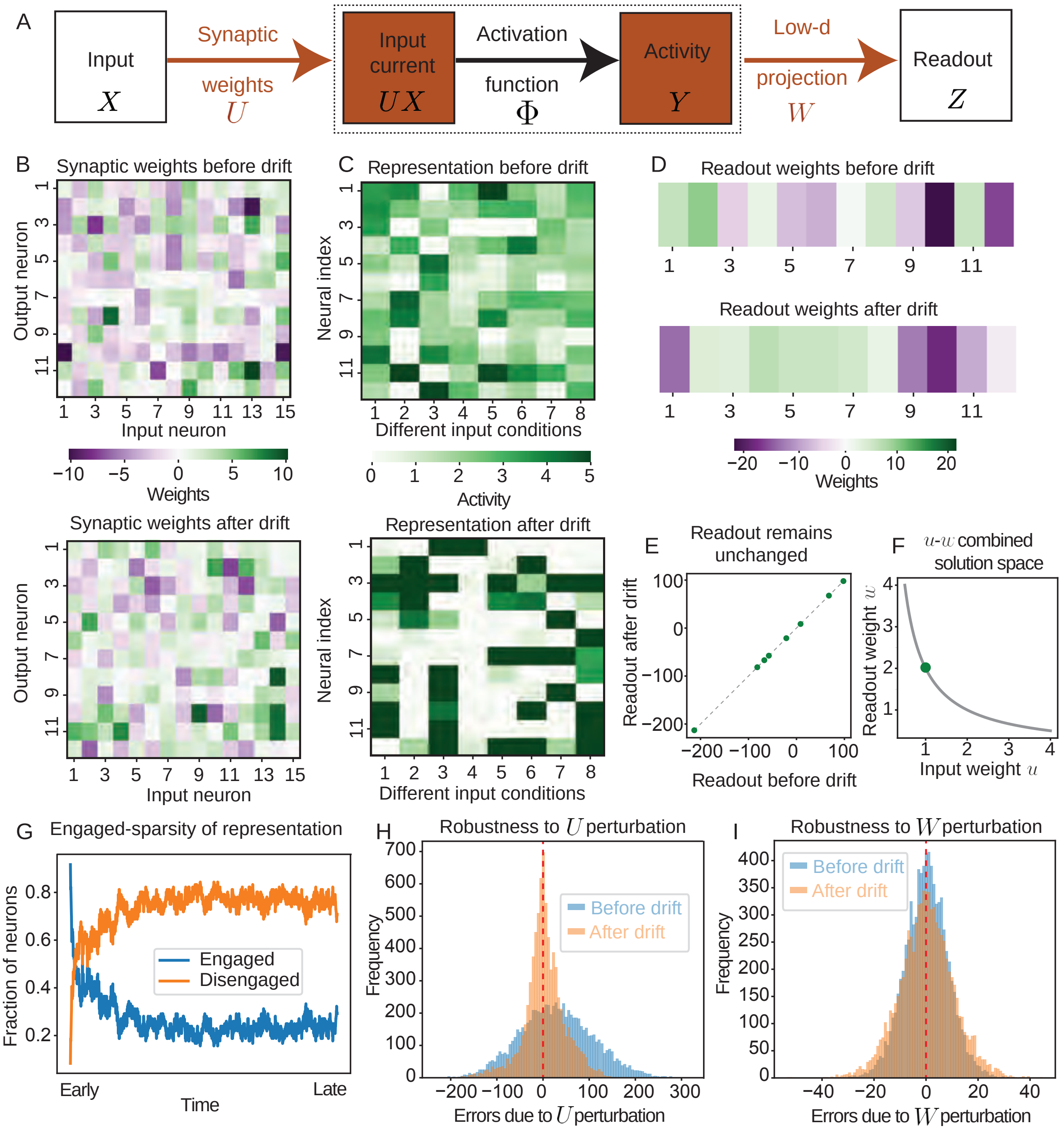


**Fig. S2.** Generalizing to dynamic readout weights. (A) Network framework with dynamic readout weights. (B) Synaptic weights before and after drift demonstrating synaptic turnover. (C) Neural activity under different input conditions before and after simulating drift. (D) Readout weights before and after drift. (E) Readout for each input condition remains the same before and after drift. (F) Toy example solution space to illustrate the need for coordinated changes in input and readout weights. (G) Fraction of engaged and disengaged (inactive and saturated) neurons over time during drift showing drift favors sparsely engaged representations. (H) Histogram of signed errors in readout due to U perturbations $\mathcal{N}(0,1)$ showing greater robustness post-drift. (I) Histogram of signed errors in readout due to W perturbations $\mathcal{N}(0,1)$ showing sparsely engaged representations don't enhance robustness to W perturbations.

While this method allowed us to generate representational changes beyond the null space of the readout weights $W$, this method may not comprehensively sample from the full combined solution space of $(U, W)$. To illustrate, consider a simplified example where a single input neuron projects with weight $u$ to a hidden layer neuron, which further projects with weight $w$ to a readout neuron. The readout is given by $z = w\Phi(ux)$, where $\Phi$ represents a clipped-threshold linear activation function and $x$ is the input. The task entails maintaining a readout $z = 2$ for an input $x = 1$, which simplifies to the constraint $w\Phi(u) = 2$. When the neuron is engaged, this further reduces to $wu = 2$, indicating that there are many solutions of synaptic and readout weight combinations, and the system should be able to diffuse in the solution space (Fig. S2F). Now, consider starting from an initial solution of $(u, w) = (1, 2)$. First, we fix $w = 2$ and attempt to change $u$ to maintain the readout. However, this leads to $2u = 2$, fixing $u$ at 1. With only one input neuron ($N_x = 1$) and one stimulus to store ($P = 1$), there are no unconstrained dimensions. Furthermore, since there are no disengaged neurons, there are no semi-constrained dimensions. The number of hidden-layer neurons ($N_y = 1$) matches the readout dimensionality ($N_z = 1$), meaning the hidden-layer activity cannot change without altering the readout. Consequently, $u$ cannot be changed. Next, we attempt to modify $w$ while keeping $u$ fixed, but this also lacks flexibility, as it requires $w = 2$. Thus, the system becomes stuck in the solution $(u, w) = (1, 2)$. However, this solution is surrounded by solutions. Exploring these requires simultaneous changes in both $u$ and $w$, which this method cannot achieve. Thus, joint modification of $u$ and $w$ is necessary to access certain solutions, highlighting a limitation of this approach.

Robustness to synaptic weight perturbations persists in this setup, but inactive and saturated neurons exhibit opposing effects when considering readout weight perturbations. Representational drift generated using this method, alternating $U$ and $W$ changes still led to sparsely engaged solutions (Fig. S2G). As before, these solutions showed enhanced robustness to input weight perturbations (Fig. S2H). Since this setup allows changes to both input synaptic weights and abstract readout weights, we also considered robustness to $W$ readout weight perturbations. The error in readout $Z$ due to a perturbation in $W$ can be quantified as $dWY$, indicating that the error is proportional to the activity level of representation-layer neurons. Neurons with no activity (i.e., inactive neurons $Y_{j,\mu} = 0$) do not contribute to the error, whereas neurons that have reached their maximum activity level (i.e., saturated neurons $Y_{j,\mu} = \alpha$) contribute the most to the error. Drift led to an increase in both inactive and saturated neurons, resulting in post-drift solutions exhibiting similar robustness to readout weight perturbations compared to pre-drift solutions (Fig. S2I). Although both engaged and saturated neurons enhanced the robustness of the model to perturbations in the input synaptic weight $U$, they had opposing effects on the robustness to changes in $W$. Therefore, when the readout weights themselves are subject to change, sparsely active solutions may offer greater robustness compared to sparsely engaged ones.

**C. Impact of stable neuronal subsets on drifting neural representations.** The activity of a subpopulation of neurons can be stabilized through various methods, without altering the core results of the paper. In our framework, we allow the responses of all neurons to change over time due to drift. However, some neural recordings suggest that a subset of neurons remains stable across time despite drift in others (1, 2). Our framework can accommodate such stable subsets of neurons through specific architectural choices. One such mechanism involves stable input synaptic weights. Neural activity results from inputs transformed through synaptic weights followed by an activation function. In our setup, we fix the inputs and activation functions. Therefore, if the synaptic weights onto a subset of neurons are also held fixed, the responses of these neurons will remain stable across time. To incorporate this possibility into our model, we divided the neural population into stable and unstable subsets. We fixed the input weights onto the stable neurons and simulated drift by allowing the weights onto the unstable population to change, as in previous simulations. As expected by design, the activity of the stable neurons (neurons 1 and 2) remains unchanged, while the unstable population exhibits drift (Fig. S3A)—yet the overall readout remains invariant (Fig. S3B). Another mechanism for producing a stable subset of neurons involves the structure of readouts. If a neuron's activity is directly readout as a cognitive or behavioral output, then that functional role can anchor its response, making it resistant to change—even if the synaptic weights onto it vary over time. In our framework, this can be modeled by including additional readouts that are tied to specific neuronal responses. In both cases, stabilizing a subset of neurons reduces the likelihood of previously engaged neurons becoming disengaged over time, resulting in slightly smaller changes in sparsity during drift. Nevertheless, as long as some portion of the population continues to drift, our core result holds: drift can still lead to sparsely engaged (Fig. S3C), noise-robust solutions (Fig. S3D).

While assuming stable input weights onto specific neurons or direct readout from only a subset of neurons may seem extreme, milder forms of these mechanisms could underlie the stable neuronal subsets observed in biology. For example, in the somatosensory cortex, neurons at the core of each whisker column are preferentially tuned to that column's whisker ("columnar-tuned") and are more likely to project to secondary somatosensory cortex. In contrast, neurons in the surround of each whisker column respond to other whiskers, and often to multi-whisker sequences ("non-columnar"). In tasks requiring discrimination of individual whisker touches, columnar-tuned neurons were found to be significantly more stable than non-columnar ones (1). This stability may arise from both anatomical constraints on synaptic connections—limiting the access of core column neurons to inputs from other whiskers—and their immediate relevance for whisker discrimination readout possibly in the secondary somatosensory cortex.

Saturation can also generate apparent stability in neural responses. A saturated neuron can still change its activity if synaptic changes reduce its input current below the saturation threshold. However, if its input current remains far above the saturation threshold, it is likely to stay saturated even after extensive synaptic changes. Such neurons may therefore appear highly stable, despite ongoing changes in the network (ex. neurons 6 and 8 Fig. S3A). This kind of stability is typically specific to a given neuron, under a particular input condition, at a time during drift when its input current is far above the saturation threshold. Nevertheless, in our model neurons with high excitability—characterized by smaller negative biases—are more likely

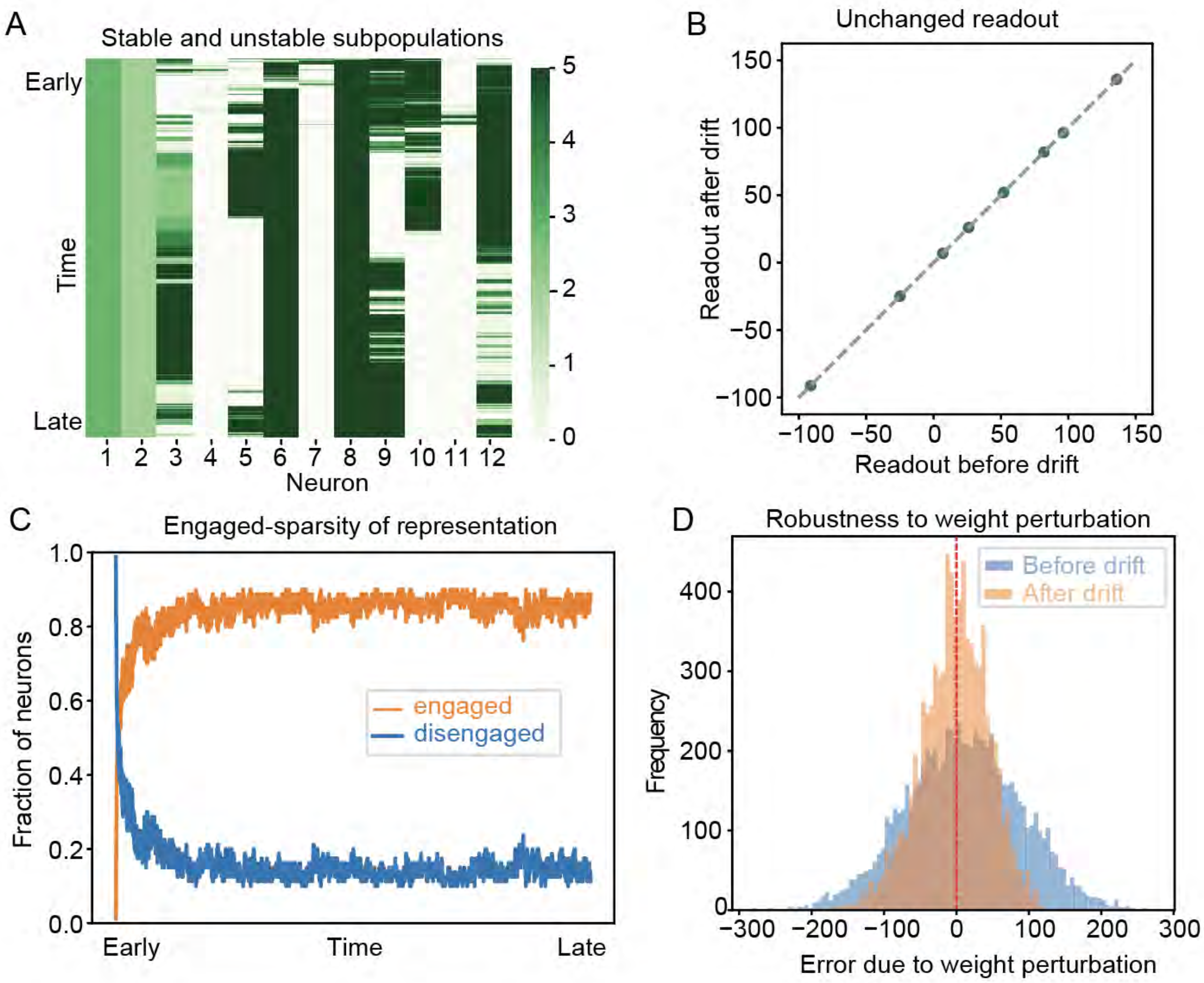


**Fig. S3.** Stable neuronal subset despite drifting of overall neural representation. (A) The activity of 2 stable and 10 unstable neurons under a particular input condition over time. (B) Readout or 1-dimensional projection of the representation before and after drift, showing that it remains the same. (C) Fraction of engaged and disengaged (inactive and saturated) neurons over time during drift showing an initial drop in the fraction of engaged neurons followed by stable maintenance. (D) Histogram of signed errors in readout due to $U$ perturbations $\sim \mathcal{N}(0, 1)$. Root-measure squared error due to weight perturbations before drift (blue) and after drift (orange).

to reach saturation across a broader range of inputs, suggesting their responses may be more consistently stable over time. Interestingly, neurons with higher excitability have indeed been observed to exhibit greater response stability (3), although it remains unclear whether this is due to saturation or other mechanisms.

## 2. Understanding the geometry and statistical properties of solution spaces

**A. Illustrative toy problem.** To illustrate why robust solutions might emerge more naturally through drift than through learning, we'll analyze the solution space and error gradients for a simple toy model. The toy model consists of a two-neuron representation $y$ (Fig. S4A), which receives input from a single sensory neuron $x$ and influences a 1-dimensional linear readout $z$. We consider a task where the system needs to reproduce a single mapping from input $x$ to output $z$.

$$z = w_1 y_1 + w_2 y_2 = w_1 \Phi(u_1 x) + w_2 \Phi(u_2 x), \tag{8}$$

where $w_1$ and $w_2$ parameterize the readout weights, $x$ is the input, $y_1$ and $y_2$ are the activities of the two neurons, and $\Phi$ (Fig. S4B) is the clipped-threshold-linear activation function given by

$$\Phi(v) = \begin{cases} 0 & \text{if } v \leq 0 \\ v & \text{if } 0 < v < \alpha \\ \alpha & \text{if } v \geq \alpha \end{cases}, \tag{9}$$

where the activity threshold is 0 and saturation threshold is $\alpha$. Between 0 and $\alpha$, changes in input currents lead to changes in neural activity, so we denote neurons in this regime "engaged". Neurons with input currents below the activity threshold 0 are inactive and those with input currents exceeding the saturation threshold $\alpha$ are saturated. Both inactive and saturated neurons exhibit activity invariance to small input current changes, thus we classify them as "disengaged". In the examples that follow, we set $\alpha = 5$, $x = 1$, $(w_1, w_2) = (1, 1)$, and analyze the solution space of $(u_1, u_2)$ for different values of readout $z$.

Synaptic weight solutions can vary in statistical properties, such as the sparsity of the representations they produce, with some level of sparsity being more prevalent than others. Suppose the required readout is $z = 4$, then the representation must satisfy $y_1 + y_2 = 4$. When both neurons are in the engaged regime (i.e., $0 < y_1 < 5$ and $0 < y_2 < 5$), we get densely engaged solutions satisfying $u_1 + u_2 = 4$ (Fig. S4C, blue line). Since flexibility along this dimension changes the neural representations, we term this as "drift dimension". At $(u_1, u_2) = (0, 4)$, the first neuron becomes inactive, so its input current only needs to satisfy $u_1 \leq 0$, giving rise to sparsely engaged solutions with one inactive and one engaged neuron (Fig. S4C orange line). Since the inequality constrains the solution space to part of a dimension, we denote this as "semi-constrained" dimension of flexibility. We also find another semi-constrained dimension $u_2 \leq 0$ when $u_1 = 4$, which also gives rise to sparsely engaged solutions. As the weight norm bound increases, larger input currents are allowed, expanding the volume of sparsely engaged solution spaces with an inactive neuron (Fig. S4C,D). In contrast, the size of the densely engaged solution space remains unchanged. For sufficiently large weight norms, the volume of sparsely engaged solution space surpasses that of densely engaged solution space, causing diffusion to favor these sparsely engaged representations.

Sparsely engaged representations are robust to noise. When a random perturbation is applied to synaptic weights $(u_1, u_2)$, it affects the input currents that neurons receive, which in turn affects the activity of engaged neurons and the readout. For inactive neurons, the activity remains at 0 if the perturbation is small enough that the input current doesn't cross the activity threshold, so these neurons don't contribute to downstream error. Therefore, a weight perturbation of the same magnitude leads to a larger error when starting from a densely engaged solution than when starting from a sparsely engaged solution (Fig. S4E,F). Saturated neurons also contribute to such increased robustness of sparsely engaged representation solutions. Small weight perturbations that do not cause the input current to fall below the saturation threshold do not affect the activity of a saturated neuron. Indeed, when the required readout is $z = 6$, we find that sparsely engaged solutions involving saturated neurons ($u_1 \geq 5, u_2 = 1$ or $u_1 = 1, u_2 \geq 5$ ) (Fig. S4G, orange lines) are more robust to weight perturbations (Fig. S4H,I) than densely engaged solutions satisfying $u_1 + u_2 = 6$ (Fig. S4G).

Different dimensional solution spaces may coexist for particular mappings. When the required readout is $z = 6$, consider a system diffusing within the densely engaged solution space, satisfying $u_1 + u_2 = 6$. When the system reaches $(u_1 = 5, u_2 = 1)$, the first neuron becomes saturated, introducing a semi-constrained dimension $(u_1 \geq 5)$. However, this flexibility is offset by a loss of drift dimension flexibility because the second neuron must now satisfy $u_2 = 1$ with no flexibility (Fig. S4G). Since a drift dimension converts into a semi-constrained dimension, the overall dimensionality of the solution space remains unchanged before and after the first neuron becomes saturated. Now, consider a different required readout of $z = 5$. Here, two types of solutions emerge: (1) Densely engaged solutions with both engaged neurons satisfying $u_1 + u_2 = 5$ (2) Maximally sparsely engaged solutions with one inactive and one saturated neuron, satisfying two inequality constraints (ex. $u_1 \geq 5, u_2 \leq 0$) (Fig. S4J). Suppose the system explores the densely engaged solution space $u_1 + u_2 = 5$ and reaches $(u_1 = 5, u_2 = 0)$. At this point, the first neuron becomes saturated and the second becomes inactive. This adds two semi-constrained dimensions ($u_1 \geq 5$ and $u_2 \leq 0$) while losing one drift dimension flexibility, increasing the dimensionality of the solution space to two. Thus, a two-dimensional maximally sparsely engaged solution space coexists with a one-dimensional densely engaged solution space.

A higher-dimensional solution space implies greater prevalence and robustness. Since maximally sparsely engaged solutions form a two-dimensional solution space, they are infinitely more numerous than densely engaged solutions, which are confined to a one-dimensional space. Furthermore, small weight perturbations in the maximally sparse solution do not alter the state: the inactive neuron remains inactive, the saturated neuron remains saturated, and both the activity and readout remain unchanged.

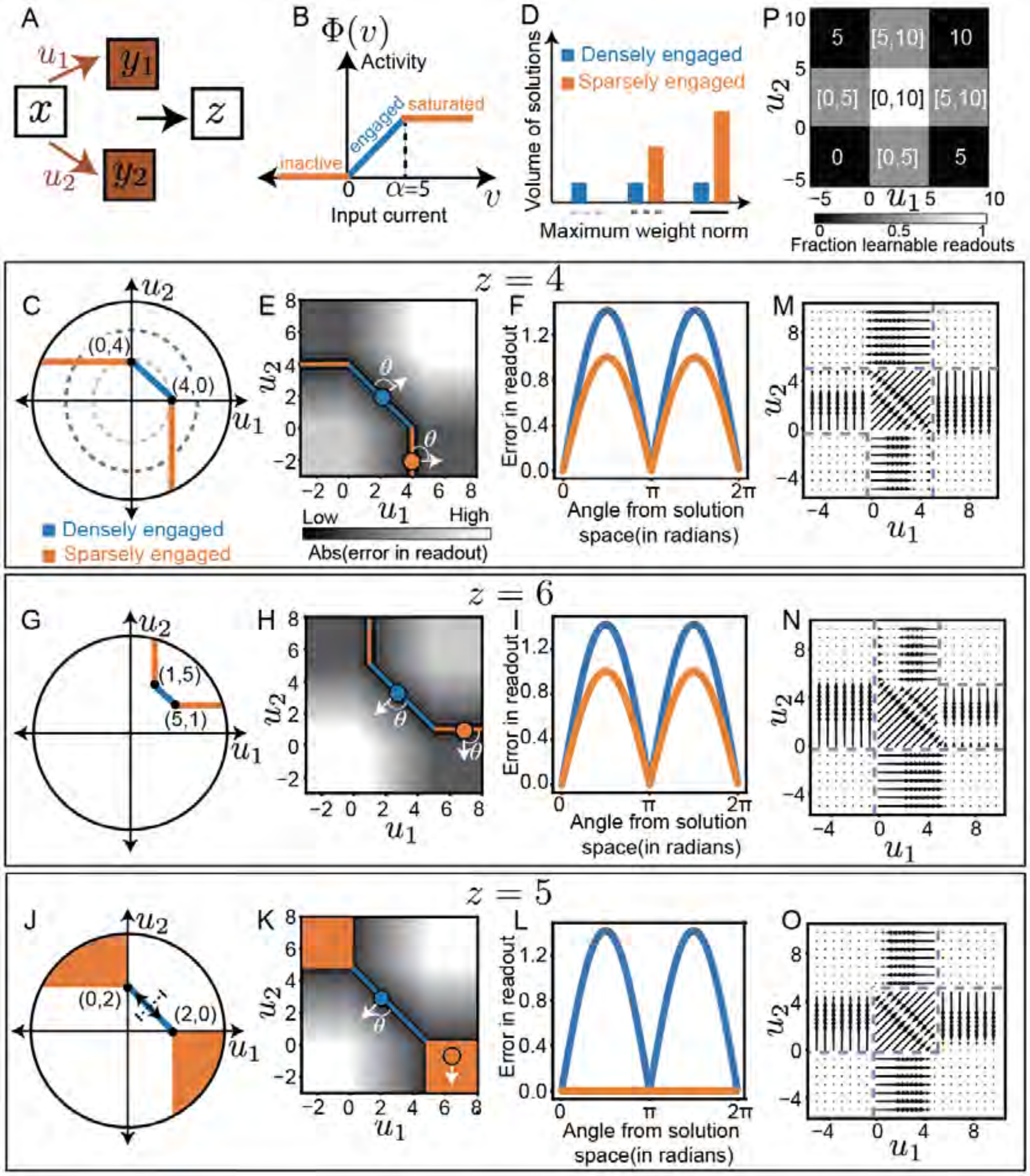

**Fig. S4.** A toy example illustrating the prevalence, robustness, and computational challenges of learning sparsely engaged solutions. (A) Network architecture for a toy example whose task is to generate a fixed readout, $z$, from two-dimensional neural activity, $(y_1, y_2)$, generated from a single sensory input, $x$, via weights, $(u_1, u_2)$. (B) Activation function $\Phi$ indicating the regime for engaged, inactive, and saturated neurons. Subsequent panels examine solutions for different readout values: $z = 4$ (C-F); $z = 6$ (G-I); and $z = 5$ (J-L). (C) Synaptic weight solution space $(u_1, u_2)$ for $z = 4$, with sparsely engaged solutions in orange and densely engaged solutions in blue. Dashed lines indicate different weight norms. (D) The volume of sparsely engaged solution space increases with increase in the weight norm bound. Line styles along the x-axis indicate the weight norm bounds shown in (C). (E) Error in readout $z$ for various $(u_1, u_2)$ and a schematic demonstrating weight perturbations (white arrow) from sparse (orange) and densely engaged (blue) solutions. (F) Weight perturbations from sparsely engaged solution $(u_1, u_2) = (4, -2)$ produces lower readout error than perturbations from a densely engaged solution, $(u_1, u_2) = (2, 2)$ across all perturbation angles $\theta$. (G,H,I) Same as (C,E,F) but with the desired readout $z = 6$, showing that sparsely engaged solutions with a saturated neuron (orange) have enhanced robustness similar to sparsely engaged solutions with an inactive neuron. Perturbations are from a sparsely engaged solution with one saturated neuron, $(u_1, u_2) = (1, 7)$ (orange dot), or a densely engaged solution, $(u_1, u_2) = (3, 3)$ (blue dot). (J,K,L) Same as (C,E,F) but with the desired readout $z = 5$, showing a 2-dimensional space of maximally sparsely engaged solutions in orange and a 1-dimensional space of densely engaged solutions in blue. Perturbations are from a maximally sparsely engaged solution with a saturated neuron and an inactive neuron $(u_1, u_2) = (7, -2)$ (orange dot), and from a densely engaged solution, $(u_1, u_2) = (2.5, 2.5)$ (blue dot). (M, N, O) Error gradient flow fields plots for desired readout $z = 4$ (M), $z = 6$ (N), and $z = 5$ (O). Arrows indicate the direction in which errors decrease, and their length corresponds to the gradient magnitude. Dashed purple lines delineate the basin of attraction reached from initialization at that location. (P) For each of the nine regions in weight space, color represents learning conduciveness, quantified as the fraction of $z$ readouts for which solution space can be reached by following the gradients. The displayed values or intervals correspond to the ranges of $z$ readouts.

This makes maximally sparse solutions not only more prevalent but also maximally robust (Fig. S4K,L). Once the system reaches this robust, higher-dimensional solution space, transitioning back to the densely engaged solution space becomes highly improbable. To do so, the system would need to shift from exploring a two-dimensional region ($u_1 \geq 5$ and $u_2 \leq 0$) to a single point ($u_1 = 0$, $u_2 = 5$), a transition that is unlikely to occur through diffusion, thereby preserving this robust representation.

These toy examples have demonstrated that sparsely engaged representations are common, implying drift will favor these solutions. Interestingly, we will see that despite being more common, these solutions are challenging to learn and can hinder subsequent learning. To do so, we will examine regions of the synaptic parameter space that facilitate learning and the types of solutions that emerge when starting from these learning-conducive regimes. Consider a scenario where the goal is to learn a mapping from an input $x$ to a desired readout $z$. A random weight configuration likely produces a readout $\hat{z}$ that differs from the desired readout $z$, resulting in a squared readout error of $e = (\hat{z} - z)^2$. To learn the mapping, the weights need to change to minimize this readout error. Learning the mapping requires adjusting the weights to minimize this error. This process is often modeled using gradient descent, where weights are updated in the direction that most effectively reduces the error—specifically, the direction opposite to the gradient of the error function ($\frac{de}{du}$). Since gradient information is essential for learning, we will analyze the gradients at different weight configurations for different required readouts $z$ while setting a fixed input $x = 1$, to identify regions of the parameter space that are most conducive to learning.

Maximally sparsely engaged initializations are unsuitable for learning because they provide no gradients. If the initial weights cause both neurons to become disengaged, small changes in input currents have no effect on neural activity, the readout, and the readout error. As a result, the error function is locally flat and lacks gradients (Fig. S4M,N,O). This prevents the weights from updating, leaving the system stuck in a local optimum regardless of the required readout. These initializations are only effective if the current readout $\hat{z}$ already matches the desired readout $z$ (Fig. S4O). For instance, when the required readout is $z = 5$ and if we initialize at $(u_1, u_2) = (-2, 7)$ which produces a maximally sparsely engaged representation $(0, 5)$, the network would already produce the correct readout 5, requiring no learning.

Partially sparsely engaged initializations face similar but less severe challenges compared to maximally sparse ones. Consider an initialization where the first neuron is engaged and the second is inactive, such as $(u_1, u_2) = (1, -4)$. If the desired readout $z$ permits a solution where the second neuron remains inactive (e.g., for $z = 4$, solutions like $(u_1, u_2) = (4, -4)$ exist), the system can leverage gradients from the engaged neuron to converge to these sparsely engaged solutions (Fig. S4M). However, if no such solution exists with the second neuron being inactive (e.g., for $z = 6$), the system initially reduces the error by increasing the activity of the first neuron. However, once the first neuron saturates, the system becomes trapped in a local optimum where both neurons are disengaged. For instance, starting from $(u_1, u_2) = (4, -4)$, the system may increase $u_1$ to reach $(u_1, u_2) = (5, -4)$. Here, the network generates neural activity of $(y_1, y_2) = (5, 0)$ and a readout error of 1. Since both neurons are disengaged, there are no gradients and the system will remains stuck (Fig. S4N). Thus, while partially sparse initializations can succeed, they frequently get stuck in local optima (Fig. S4P), making them less conducive for effective learning.

Densely engaged initializations are the most conducive to learning but tend to yield non-robust solutions. For example, consider an initialization at $(u_1, u_2) = (1, 1)$, where both neurons are engaged. For any readout within the feasible range $(0 \leq z \leq 10)$, this and other densely engaged initializations can reliably find a solution (Fig. S4M-P). However, while densely engaged initializations succeed in finding solutions, they often converge to non-robust, densely engaged solutions that are closer in parameter space, even when robust, sparsely engaged solutions are more prevalent. For instance, if the initialization is $(u_1, u_2) = (1, 1)$ and the desired readout is $z = 4$, gradient-descent learning would follow the trajectory of maximal error reduction along the line $u_1 = u_2$ ultimately reaching the solution space at $(u_1, u_2) = (2, 2)$ (Fig. S4E, left). This solution, however, is densely engaged and non-robust. Consequently, a mechanism like drift may be advantageous in guiding the system from densely engaged, non-robust solutions that are easy to learn to sparsely engaged, robust solutions that are more common.

To summarize, these toy examples suggest that given a sufficiently large weight norm, sparsely engaged, noise-robust solutions are more common and thus preferred by drift. Moreover, these robust solutions are harder to reach through learning when starting from learning-conducive regimes, suggesting that drift following learning may help find these solutions. The next few subsections will investigate the prevalence and robustness of drifting representations.

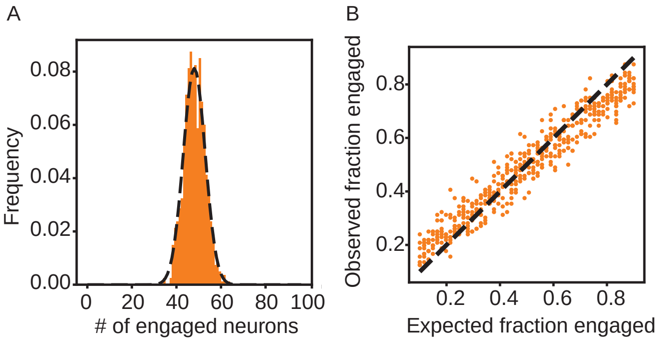


**Fig. S5.** Understanding the sparsity of solutions achieved by drift. (A) The orange histogram shows the frequency of representations having a certain number of engaged neurons $E$ during drift with input current range parameters $c_1 = -2.5$ and $c_2 = 7.5$. To avoid correlated data, we sampled only once every 500 steps and began sampling after 5000 timesteps to ensure equilibration. The dashed line represents the theoretical prediction for the probability of representations with different number of engaged neurons given by $B(N_y P = 96, p_{\mathcal{E}} = 0.5)$, demonstrating a good match between the theoretical sparsity prediction and the sparsity of representations explored by drift. (B) For 50 simulations of drift with different $c_1$ and $c_2$ values chosen to explore a wide range of engaged neuron fractions ($0.1 \leq p_{\mathcal{E}} \leq 0.9$), the x-axis shows the expected fraction of engaged neurons, while the y-axis shows the observed fraction of engaged neurons. Sampling was performed every 500 steps for each simulation to avoid correlated data.

 

**B. Understanding the prevalence of sparsely engaged solutions.** Here, we seek to better understand the relationship between solutions explored by drift and their prevalence. As weight norm bounds increased, we observed that drift-explored representations became more sparsely engaged and robust. We hypothesize this occurs because larger weight norm bounds enable larger input currents, expanding exploration ranges for inactive and saturated neurons, and consequently increasing the prevalence of sparsely engaged solutions. To validate this hypothesis, we directly impose input current limits instead of weight norm bounds and compare the sparsity of drift-explored representations to their expected solution prevalence. To simplify prevalence computation, we approximate solution frequency with the overall representation frequency at different sparsity levels, irrespective of solution status.

We analyze the same model configuration, consisting of $N_x$ input neurons, $N_y$ neurons in the representation layer, and $N_z$ abstract readouts. The task involves maintaining a mapping between input $X_\mu$ and corresponding readout $Z_\mu$ for $\mu = 1, .., P$ mappings. The weights $U$ onto representation-layer $Y$ are plastic and the readout is defined by a fixed matrix $W$. Consider limiting the input current range to $(c_1, c_2)$, where $c_1 \leq 0$ and $c_2 \geq \alpha$. Under these conditions, a neuron is engaged with probability $p_\mathcal{E} = \frac{\alpha}{c_2 - c_1}$. Consequently, the number of engaged neurons, $E = \sum_{\mu=1}^{P} |\mathcal{E}^\mu|$, is binomially distributed as

$$E \sim B(N_y P, p_\mathcal{E}) \implies P(E = k) = \binom{N_y P}{k} p_\mathcal{E}^k (1 - p_\mathcal{E})^{N_y P - k}. \quad [10]$$

For instance, if $c_1 = -2.5$ and $c_2 = 7.5$ then the probability of being engaged and disengaged are the same at 0.5. Here, we observed an excellent match between this theoretical distribution from Eq. 10 and the fraction of time points during drift that the system spent in representations with varying numbers of engaged neurons (Fig. S5A). By systematically varying $c_1$ and $c_2$ over the ranges $-22.5 \leq c_1 \leq -0.27$ and $27.5 \leq c_2 \leq 5.27$, we modulated drift to favor different sparsity levels from 0.1 to 0.9 (Fig. S5B). The expected fraction of engaged neurons, $p_\mathcal{E}$, closely matched the observed mean fraction during drift, with minor discrepancies at extremely low or high sparsity. These findings support that sparsely engaged representations are favored by drift due to the inherent prevalence of these representations when input currents are permitted to be large.

While sparsely engaged solutions might have been favored due to higher-dimensionality of their solution spaces, in our simulations this is not the driving factor. Let there be $N_z + n_\mathcal{E}$ engaged neurons for a particular input condition $\mu$, where $N_z$ is the dimensionality of the required readout. Since the number of engaged neurons is $n_\mathcal{E}$ greater than the readout dimensionality, the engaged submatrix $W^\mu$ has a $n_\mathcal{E}$-dimensional null space, implying there are $n_\mathcal{E}$ drift dimensions for $\mu$. During solution space exploration, if $n_\mathcal{E} + 1$ of those engaged neurons simultaneously become disengaged then $n_\mathcal{E} + 1$ semi-constrained dimensions are added. However, there were only $n_\mathcal{E}$ drift dimensions for $\mu$ before, so only $n_\mathcal{E}$ drift dimensions can be lost. Thus, the new solution space would become larger by one dimension compared to the previous solution space. In our drift simulations, for $N_z = 1$, this would require zero engaged neurons for an input condition. However, we observed no cases where the number of engaged neurons dropped to zero, suggesting that increased dimensionality of solution space was not responsible for exploration of sparsely engaged solutions.

Higher-dimensional solution spaces not being explored in our simulations raises intriguing questions about their feasibility and accessibility. As illustrated in the toy example in Fig. S4, such higher-dimensional solution spaces arise only under highly specific conditions involving precise values of the readout, readout weights, and activity thresholds. It is plausible that, similar to some other toy examples explored with required readouts of $z = 4$ and $z = 6$, the input-output mappings in our simulations may not have had higher-dimensional solution spaces. Such higher-dimensional solution spaces may become more feasible if we allow non-zero readout errors, changes in readout weights, and dynamic activity and saturation thresholds. Alternatively, such higher-dimensional solutions may indeed exist but could be inherently more challenging to reach with our current methods or through diffusion-driven exploration in general. Future work could leverage tools from statistical mechanics to rigorously quantify the volume of solution spaces across varying sparsity levels. This may help determine whether such high-dimensional solution spaces exist and offer insights into how they might be accessed, potentially enabling exceptional robustness and stability.

**C. Understanding the robustness of sparsely engaged solutions.** Here, we delve deeper into the relationship between robustness and representational sparsity. Consider applying a weight perturbation $dU$; the new downstream readout for the $\mu^{th}$ stimulus then becomes $\hat{Z_\mu} = \sum_i W_i \Phi\left(\sum_j (U_{i,j} + dU_{i,j}) X_{j,\mu}\right)$. The readout error $\hat{Z_\mu} - Z_\mu$ is given by

$$err = \hat{Z_\mu} - Z_\mu = \sum_{i=1}^{N_y} W_i \left( \Phi\left(\sum_{j=1}^{N_x} (U_{i,j} + dU_{i,j}) X_{j,\mu}\right) - \Phi\left(\sum_{j=1}^{N_x} U_{i,j} X_{j,\mu}\right)\right). \quad [11]$$

Assume $dU$ is sufficiently small such that none of the neurons cross thresholds. Then for $i \notin \mathcal{E}^\mu$ (i.e., disengaged neurons), we have $\Phi\left(\sum_{j=1}^{N_x} (U_{i,j} + dU_{i,j}) X_{j,\mu}\right) = \Phi\left(\sum_{j=1}^{N_x} U_{i,j} X_{j,\mu}\right)$, so these neuron-stimuli $(i, \mu)$ pairs don't contribute to the readout error. Since we assume engaged neurons remain engaged after perturbation, we can remove the non-linearity to get

$$err = \sum_{i \in \mathcal{E}^\mu} W_i \left(\sum_{j=1}^{N_x} dU_{i,j} X_{j,\mu}\right). \quad [12]$$

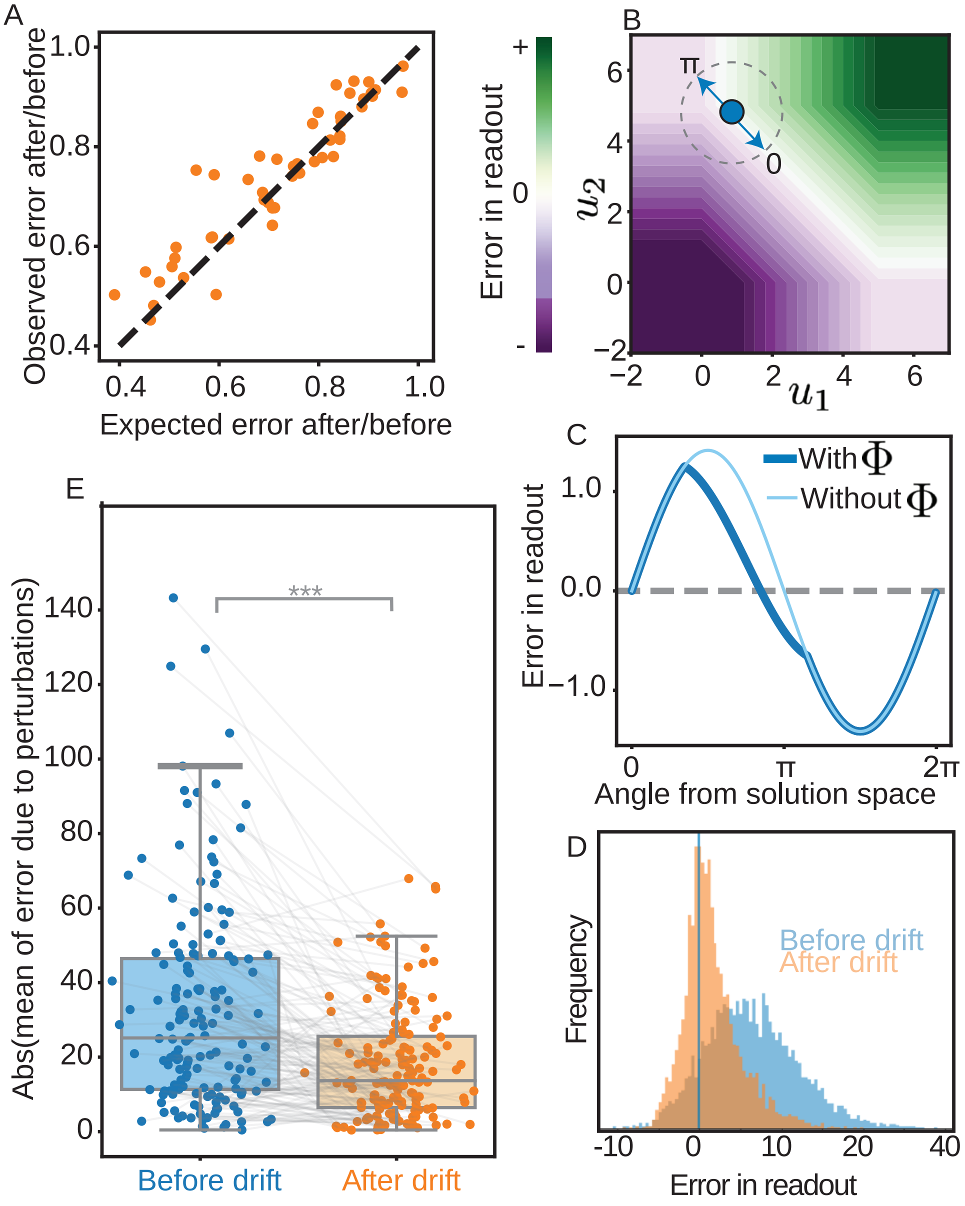


**Fig. S6.** Understanding the robustness of solutions achieved by drift. (A) The x-axis represents $\sqrt{\beta}$, the expected ratio of errors after drift to before drift, while the y-axis shows the observed fraction of errors after drift compared to before drift for each of the 50 simulations from (Fig. S5B). (B) Toy example with $z_\mu = 6$. Signed error in readout $z$ for various $(u_1, u_2)$ values with a schematic demonstrating weight perturbations in two opposite directions. The arrow towards $\pi$ shows perturbation in the direction of threshold crossing (C) Signed error in readout due to a perturbation of size 1 from densely engaged solution $(u_1, u_2) = (1.3, 4.7)$ with the second neuron's activity $y_2 = 4.7$ very close to the saturation threshold $\alpha = 5$. The dark blue line indicates signed errors in a regular network with $\Phi$ non-linearity, showing an asymmetry with less positive errors and more negative errors. While the light blue line indicates symmetric errors in the absence of non-linearity. (D) Histogram of errors due to weight perturbations $dU \sim \mathcal{N}(0, 1)$ in blue for the initially learned solution, and in orange for the solution after drift, showing mean centering of the error histogram through drift. (E) Mean of unsigned error due to weight perturbations for each of the 8 input conditions for 20 different simulations before drift and after drift. n=160, p<0.001***, Wilcoxon test.

If $\beta$ is the fraction of engaged neurons for input condition $\mu$ then $i \in \mathcal{E}^\mu$ only sums over $N_y\beta$ neurons. If $W$, $dU$, and $X$ are uncorrelated random variables with standard deviations $\sigma_W$, $\sigma_{dU}$, and $\sigma_X$ respectively, then the standard deviation of the error due to perturbation is given by

$$std(err) \approx \sqrt{N_y \beta N_x}\,\sigma_X \sigma_W \sigma_{dU}. \qquad [13]$$

Drift promotes sparsely engaged solutions, characterized by fewer engaged neuron-stimulus pairs (lower $\beta$), leading to a reduced standard deviation of errors. By exploring solutions with varying sparsity $\beta$ through limiting input currents during drift, we found a strong correlation between the ratio of standard deviation of errors before and after drift and the ratio of square root of the fraction of engaged neurons before and after drift, as predicted by Eq. 13 (Fig. S6A). This suggests that the robustness of post-drift solutions arises from sparsely engaged neurons maintaining their activity levels despite weight perturbations.

In our previously analysis, we assumed that the perturbation $dU$ doesn't lead to threshold crossing, although this assumption may not always hold. If the perturbation is sufficiently large or the input current is close to the thresholds, then weight perturbations may cause neurons to cross thresholds. When an engaged neuron crosses threshold to become disengaged, the weight change onto that neuron until the threshold crossing contributes to an error, while the remaining perturbation onto that neuron doesn't alter its activity, and thus doesn't contribute to errors. Consider the previously described toy model with a required readout $z = 6$ and a particular weight solution of $(u_1, u_2) = (1.3, 4.7)$ (Fig. S6B). We find that perturbations

from this solution, towards threshold crossing produce smaller positive errors than expected for a network without threshold non-linearity. Further, at threshold crossing, the solution space dimensions change, in this case from $(u_1 + u_2 = 5)$ to $(u_2 \geq 5)$, so a perturbation along the engaged solution space $(u_1 + u_2 = 5)$ at angle $\pi$ ends up producing negative errors. Consequently, the error due to perturbations are not symmetric around 0, in this case producing fewer positive errors than negative errors (Fig. S6C). Which in turn manifests as a bias in the error, producing asymmetric error histograms (Fig. S6D).

Solutions before drift show more bias in errors due to perturbation than solutions after drift. Crossing of the threshold becomes more likely when the input current is close to one of the thresholds, which in turn occurs more often when input drive exploration range is small. In our simulations where weight norms are allowed to be sufficiently large and no constraints are imposed on the input currents, disengaged neurons have a very large exploration range, while engaged neurons always have a small exploration range $(0, \alpha)$. As a result, engaged neurons are more likely to cross the threshold and become disengaged than disengaged neurons are to become engaged. Since initially learned representations have many more engaged neurons, while representations explored by drift have far fewer engaged neurons, such asymmetrical error histograms are relatively more common for learned representations than for representations explored by drift. Thus if the initially learned solution produces an asymmetrical error histogram, drift tends to remove this bias by shifting the mean of the histograms closer to 0 (Fig. S6D,E).

## 3. Relation to Biology/Experiments

**A. Decorrelation of synaptic weights and neural representations over time.** The rate of representational turnover may change over time. As we model drift as diffusion in the synaptic weight solution space, synaptic weights at neighboring time points are correlated. Over longer periods, this correlation gradually decreases and equilibrates (Fig. S7A). The decorrelation of synaptic weights corresponds to a parallel decorrelation of neural representations over time (Fig. S7B). However, not all synaptic weight changes result in changes to neural activity. Of the three types of flexibility—unconstrained dimensions, semi-constrained dimensions, and drift dimensions—only the drift dimensions driven by engaged neurons lead to representational changes. While learning generates densely engaged representations, early drift induces sparsification, transforming drift dimensions into semi-constrained dimensions. Consequently, early in the drift process, most synaptic changes produce representational changes. In contrast, after equilibration, when many neurons are disengaged, more synaptic changes occur without affecting representations, reducing the rate of representational change. In our simulations, diffusion in the weight space results in comparable rates of synaptic weight changes during both early and later drift (Fig. S7C). However, representations change more rapidly during early drift than later drift (Fig. S7D). This phenomenon is consistent with experimental findings, as the rate of representational changes shows an initial decrease. While it is unclear whether the greater initial representational changes are solely due to learning or also due to drift, our model predicts that even after learning is completed, the rate of representational drift may differ, with greater drift occurring soon after learning and less drift later.

**B. Exploring representations with many inactive neurons through drift.** Drift can also favor sparsely active representations. Previously, we drew inputs $X_{i,\mu}$ from $\mathcal{N}(0,1)$, allowing for both positive and negative values, which resulted in input currents being equally likely to be positive or negative. Additionally, we simulated representational drift in networks with no bias or baseline activity ($B$ was a zero matrix in $Z = W\Phi\,(UX + B)$) and set the activity threshold at 0. Under these conditions, the balance of positive and negative input currents resulted in neurons being equally likely to be inactive or active (engaged or saturated). Since we allowed very large weight norms, the exploration range was greater for saturated neurons compared to engaged neurons. Consequently, most active neurons were saturated, and the representations were sparsely engaged, consisting predominantly of inactive and saturated neurons. However, adjusting these parameters can reduce the likelihood of saturated neurons. For instance, when inputs $X$ are restricted to non-negative values and paired with a negative bias or baseline, the exploration range for inactive neurons increases, while that for saturated neurons decreases. Additionally, increasing the saturation threshold $\alpha$ and imposing smaller weight norm bounds further reduce the prevalence of representations with saturated neurons.

To favor inactive neurons, we configured $B$ as a constant matrix with a value of -15, initialized weights from $U \sim \mathcal{N}(0, 0.5)$, enforced smaller weight norm bounds, and defined the activity and saturation thresholds as 0 and 10, respectively. This configuration allowed drift to extensively explore large negative input currents (Fig. S8A) with smaller changes in weight distribution (Fig. S8B). While most neurons were active before drift, only a few remained active afterward (Fig. S8C,D). These representational changes occurred despite stable readouts (Fig. S8E). Drift led to an increase and eventual plateau in the number of inactive neurons, a decrease in the number of engaged neurons, and a consistently low number of saturated neurons (Fig. S8F,G) producing sparsely active representations more akin to biological systems. Like sparsely engaged solutions, these sparsely active representations also showed robustness to synaptic weight changes $U$ (Fig. S8H). In addition, they also showed robustness to changes in readout weights $W$ as error due to $W$ changes is proportional to activity levels (Fig. S8I). In these simulations, we observed a variety of single-neuron behaviors such as intermittent firing (Fig. S8J, input condition 8), relatively stable firing at a particular input condition (Fig. S8K, input condition 7), field switching between input conditions (Fig. S8K, input conditions 3 and 4), and gain or loss of fields (Fig. S8L, input conditions 1 and 6). Neurons also exhibited heterogeneous firing rates: some cells fired at high rates across multiple input conditions (Fig. S8K), while others that remained less active (Fig. S8J,L).

**C. Drift helps decorrelate neural representations across different input conditions.** Solutions explored by drift can provide additional advantages, such as better separability of neural representations. In biology, neural representations for two different

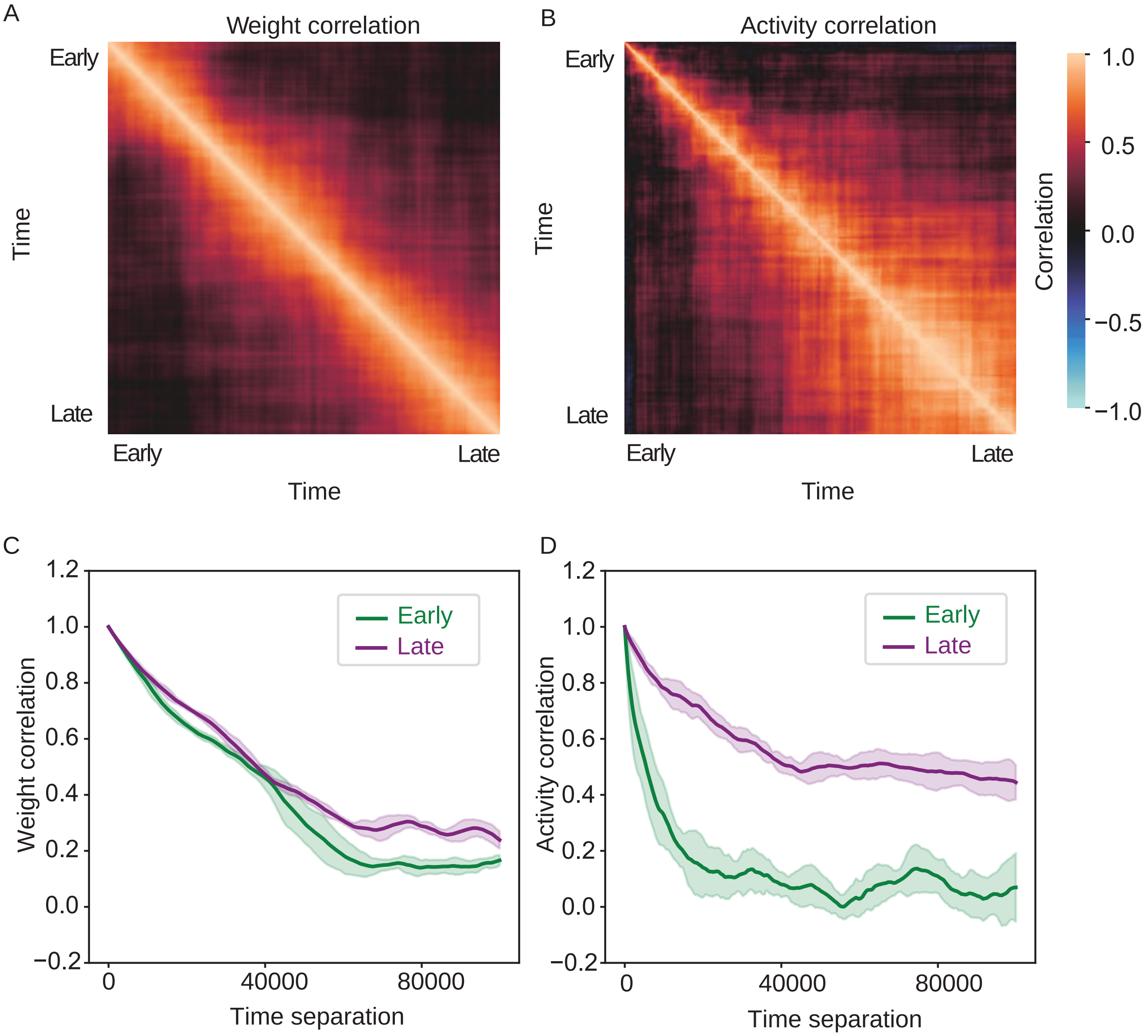


**Fig. S7.** Decorrelation of synaptic weights and neural representation. (A) Heatmap of the correlation between synaptic weights at different timepoints during drift, illustrating a gradual decorrelation over time. (B) Heatmap of the correlation between neural representations for all 8 input conditions at different timepoints, showing an initial rapid decorrelation followed by a relatively slower rate of decorrelation. (C) Lines depict the correlation between average synaptic weights during the first 10,000 timepoints of early drift (green) and during a 10,000-timepoint interval at a later stage of drift, compared to weights at 100,000 timepoints. Shaded area covers one standard deviation away from the mean. (D) Similar to (C), but showing the decorrelation of neural activity with faster decorrelation during early drift and a slower rate of decorrelation in the later stages of drift.

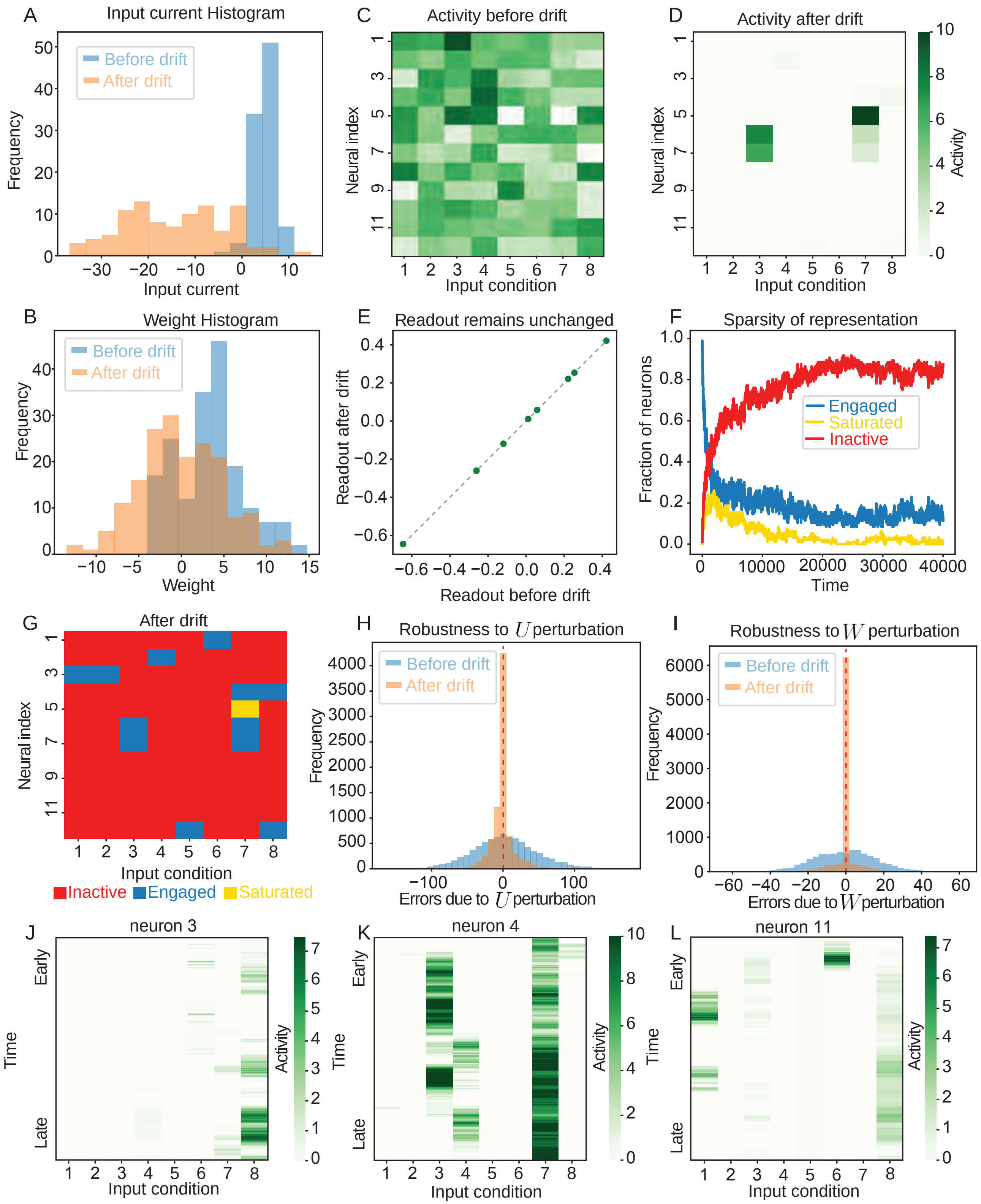


**Fig. S8.** Reaching sparsely active solutions through drift. (A) Histogram of input current before (blue) and after drift (orange), showing exploration of negative input currents after drift. (B) Histogram of weights before (blue) and after drift (orange) showing relatively stable weight distribution. (C) Neural representation under different input conditions before drift is densely active. (D) Neural representation after drift is sparsely active. (E) Readout before and after drift remains the same. (F) Fraction of engaged, inactive, and saturated neurons during drift, showing increase in the number of inactive neurons. (G) Inactive, engaged, and saturated neurons after drift. (H) Errors due to perturbation of synaptic weights $U$, showing drift improves robustness to U changes. (I) Errors due to perturbation of readout weights $W$, showing drift improves robustness to $W$ changes when exploring sparsely active solutions. (J-L) Three example neurons' activities under different input conditions over time during drift showing relatively stable fields, intermittent firing, gain, loss, and switching of fields.

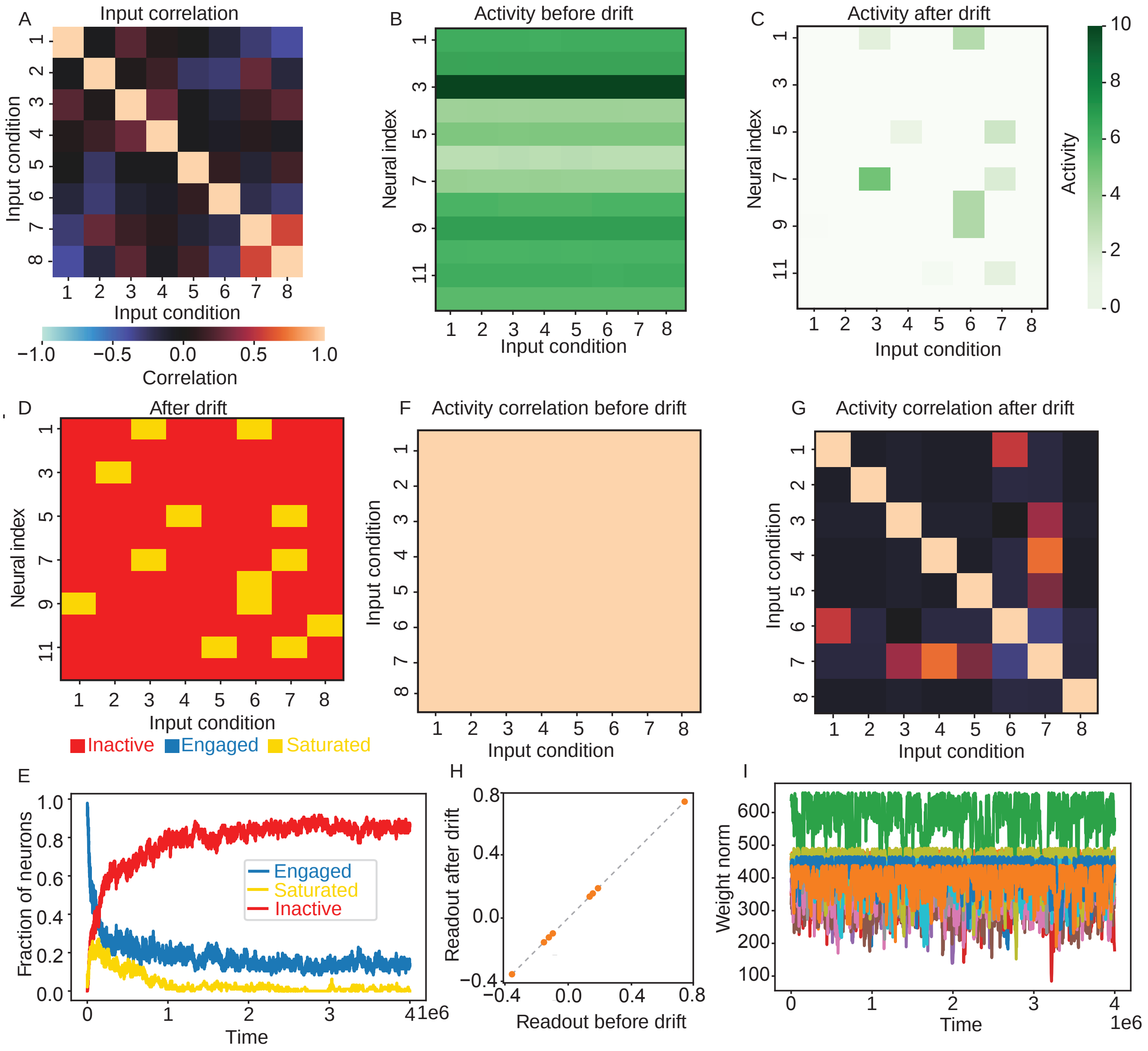


**Fig. S9.** Decorrelation of representations across different input conditions. (A) Correlation across $P = 8$ different input conditions. (B) Neural representations under these $P = 8$ input conditions after gradient descent (before drift), showing they are very similar due to correlated $\eta$ allocation. (C) Sparsely active neural representations after drift. (D) Inactive, engaged, and saturated neurons after drift. (E) Fraction of engaged, inactive, and saturated neurons during drift, showing an increase in the number of inactive neurons. (F) Correlation of neural activity across different input conditions after learning, showing the representations are highly correlated. (G) Correlation of neural activity across different input conditions after drift, demonstrating that drift has decorrelated the representations. (H) Readout before and after drift, showing maintenance of readouts. (I) Weight norms for each neuron (different colors) over time during drift, initialized with smaller weights to prevent large input currents.

 

input conditions can be highly correlated when memories are encoded close in time, as the same neurons are excitable and thus may get incorporated into both representations. However, if animals need to distinguish between these two different inputs, correlated representations pose a challenge. They are not easily separable, require carefully chosen decision boundaries for classification, and are not robust to noise. In contrast, orthogonal representations for different input conditions enhance their separability. If learned representations for distinct inputs are correlated, orthogonalizing them could be beneficial. Drift can naturally lead to orthogonal representations because, in high-dimensional spaces, there are many more orthogonal vector pairs than correlated ones. This implies that the representations explored for different input conditions are likely to become decorrelated. To model this phenomenon, we used a network with a constant bias/baseline of $-15$, and initialized the wights with a smaller standard deviation $U \sim \mathcal{N}(0, 0.5)$, to keep the weight norms low and avoid saturated neurons. During allocation, instead of randomly choosing $\eta$ for each $\mu$, we generated highly correlated $\eta_\mu$, and then performed gradient descent to learn the mappings from inputs to readouts for each $\mu = 1, ...., P$. Though the inputs were decorrelated (Fig. S9A), the learned neural representations for different input conditions $\mu$ were highly correlated (Fig. S9B, F). Upon drifting we found that the system explored sparsely active representations that became decorrelated across different input conditions (Fig. S9C-G), while maintaining fixed readouts and relatively stable weight norms (Fig. S9H,I). This suggests that mere random exploration of solution space can help the system by finding decorrelated representations that improve separability.

**D. Partial observability can obscure stable low-dimensional structure.** In this paper, we assume that certain cognitive or behavioral readouts remain stable over time, even as the underlying neural representations change. We further assume that these stable readouts are determined by a fixed function of neural activity. For simplicity, we focus on a single readout, $Z$, modeled as a linear projection of the neural population activity $Y$ (Fig. S10A). Interestingly, experimental studies have often failed to identify stable linear decoders that remain valid despite representational drift, leading to the hypothesis that downstream readouts may be dynamically changing (4). While our modeling framework also allows for drift with changing readouts (see SI Appendix 1B), here we ask a different question: could an existing stable linear projection be difficult to detect in experimental data? Specifically, although many neurons may contribute to a given representation and influence downstream regions, experiments often record activity from only a small subset of neurons. Thus, we ask whether such partial observability can obscure the existence of a fixed linear readout, even when one is present.

We first assess whether a stable decoder can be identified when we have access to all neurons. As expected under this setup, training a linear regression model on neural activity early in the drift allows accurate prediction of the readout even at later stages (Fig. S10B, red line). This is because the regression model effectively recovers the same readout weights used to generate the drift (Fig. S10C). These weights define a 1-dimensional subspace onto which the high-dimensional neural activity is projected. Under each input condition, the neural representation lies on a point manifold in this readout space, and this location remains invariant over time despite drift. As a result, initial, intermediate, and final representations for a given input condition all map to the same point along the readout axis (Fig. S10D).

This readout invariance becomes harder to detect when only a subset of the neural population is observed. Next, we train a linear regression model to predict the 1-dimensional readout using activity from only half the neurons (e.g., 6 out of 12) early in drift (Fig. S10A, bottom), the model already performs poorly—even at the training timepoints (Fig. S10B, dark green line). This occurs because no linear mapping exists within the observed neuronal subspace that can reproduce the true readout. As drift progresses, the model's performance deteriorates further. Even when trained on data spanning all timepoints, the regression model yields lower accuracy (Fig. S10B, dotted green line), and the recovered weights fail to align with the true readout weights for the subsampled neurons (Fig. S10C). Consequently, the representations corresponding to a given input condition no longer project to a fixed point manifold along the readout axis (Fig. S10D). This suggests that the low-dimensional structure imposed by the fixed readout becomes obscured when only a partial neural population is accessible.

## 4. Method for Constraining Weight Norms During Drift

To curtail synaptic weights $U$ from becoming excessively large, we introduced a constraint that limits the magnitude of weights associated with each representation-layer neuron $j$, such that $\sum_{i=1}^{N_x} U_{j,i}^2 \leq (U_{max_j})^2$, where $U_{max_j}$ is the maximum weight norm allowed for neuron $j$, which allows heterogeneity across neurons but maintains stability over time. After every learning event, we set $(U_{max_j})^2$ to be $\sum_{i=1}^{N_x} \left(U_{j,i}^{gd}\right)^2 + 5$, where $U^{gd}$ is the learned synaptic weight matrix. When a proposed change $\delta\eta$ caused the weights onto a representation-layer neuron $j$ to exceed its weight norm bound $(\sum_{i=1}^{N_x} U_{j,i}^2 > (U_{max_j})^2)$, we adjusted the change so that the weight norm exactly equals the bound $(U_{max_j})^2$. However, in this process, we still need to ensure that the readouts are preserved. To achieve this, we scaled the proposed change $\delta\eta_{j,\mu}$ by $\gamma_j$ only when $j \notin \mathcal{E}^\mu$, so that the activity $Y_{j,\mu}$ and thus the readout $Z_\mu$ remain the same. The new $\eta$ at time $t+1$ is given by

$$\eta_{j,\mu}^{t+1} = \begin{cases} \eta_{j,\mu}^t + \delta\eta_{j,\mu}, \text{if } j \in \mathcal{E}^\mu \\ \eta_{j,\mu}^t + \gamma_j \delta\eta_{j,\mu}, \text{ otherwise} \end{cases}, \quad [14]$$

Since, changes can only be made in specific $\eta_{j,\mu}$, we first write the weight norm in terms of $\eta_{j,\mu}^{t+1}$

$$\sum_{i=1}^{N_x} U_{j,i}^2 = \sum_{i=1}^{N_x} \left( \sum_\mu \eta_{j,\mu}^{t+1} X_{ext_{\mu,i}}^{-1} \right)^2. \quad [15]$$

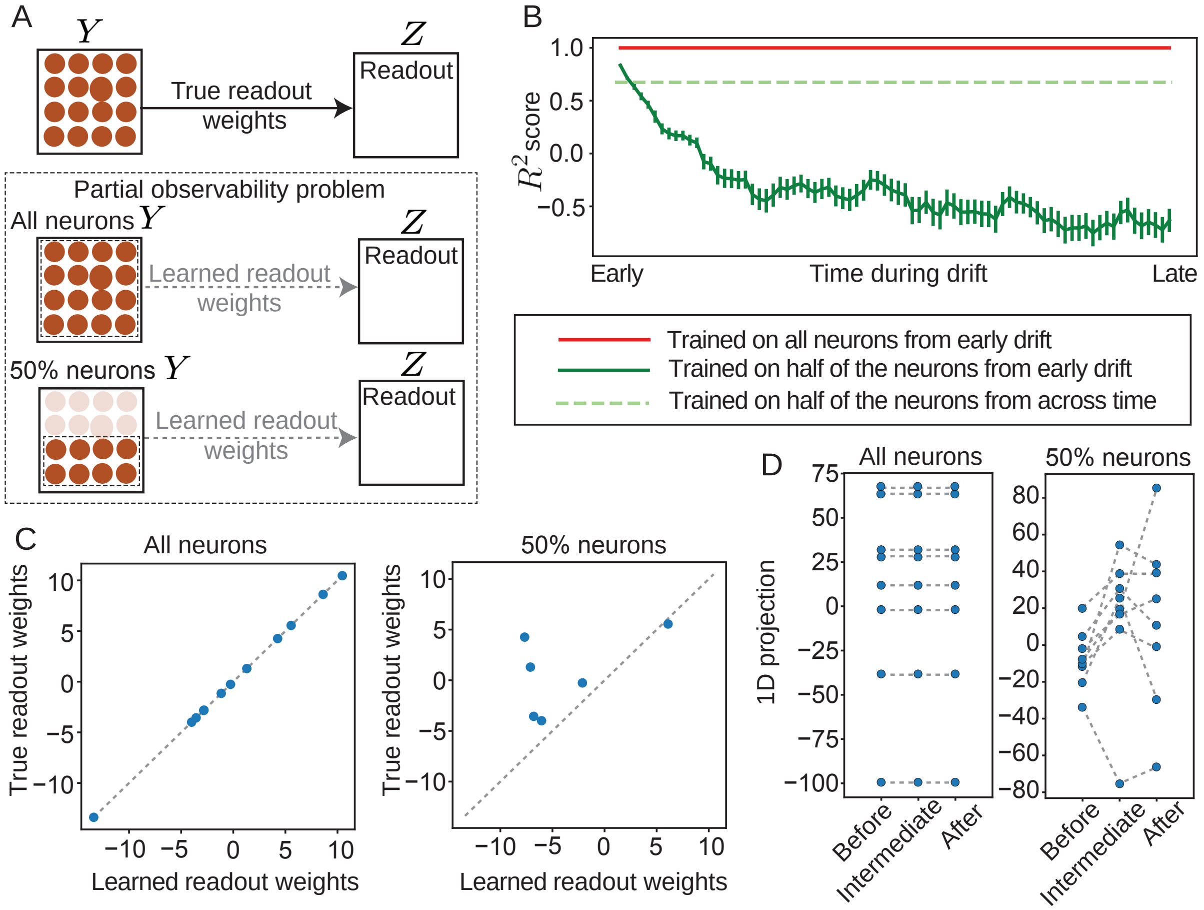


**Fig. S10.** Partial observability can obscure stable low-dimensional structure, even when it is known to exist. (A) Schematic illustrating the existence of a linear projection from the full $Y$ representation to the $Z$ readout, and posing the question of how partial observability of neurons impacts the ability to linearly decode $Z$. (B) $R^2$ score for predicting the readout $Z$ from neural representation $Y$, evaluated at different time points during drift. The solid red line shows performance when the model is trained on all neurons in the $Y$ layer using data from early drift. The solid green line shows performance when trained on a randomly selected subset comprising half of the neurons (6 out of 12) from early drift. The dotted green line shows performance when trained on half of the neurons using samples drawn from various time points throughout drift. (C) The true readout weights used to generate drift plotted against the readout weights learned by regression with all neurons (Left) and half of the neurons (Right). (D) One-dimensional projections onto linear regression weights for a fixed input condition across early, intermediate, and late stages of drift. Left: projections using weights trained on all neurons. Right: projections using weights trained on a randomly selected subset of half the neurons.

For the updated weights at time $t+1$, we want the weight norm to be $(U_{max_j})^2$. Additionally, to target changes specifically in the non-engaged dimensions of $\eta$, we separate the contribution of $\mu$s' based on whether $j \in \mathcal{E}^{\mu}$. As a result we obtain

$$
\begin{aligned}
(U_{max_j})^2 = \sum_{i=1}^{N_x} \Bigg( \Big( \sum_{\mu, j \in \mathcal{E}^{\mu}} (\hat{\eta}_{j,\mu}) X^{-1}_{ext_{\mu,i}} \Big)^2 + \Big( \sum_{\nu, j \notin \mathcal{E}^{\nu}} (\eta_{j,\nu} + \gamma_j \delta\eta_{j,\nu}) X^{-1}_{ext_{\nu,i}} \Big)^2 \\
+ 2 \Big( \sum_{\mu, j \in \mathcal{E}^{\mu}} (\hat{\eta}_{j,\mu}) X^{-1}_{ext_{\mu,i}} \Big) \Big( \sum_{\nu, j \notin \mathcal{E}^{\nu}} (\eta_{j,\nu} + \gamma_j \delta\eta_{j,\nu}) X^{-1}_{ext_{\nu,i}} \Big) \Bigg),
\end{aligned}
\tag{16}
$$

where $\hat{\eta} = \eta^t_{j,\mu} + \delta\eta_{j,\mu}$ is the new $\eta$ with the proposed change $\delta\eta$. Rearranging terms from 16, we get a quadratic equation in $\gamma_j$:

$$
\begin{aligned}
0 = & \sum_{i=1}^{N_y} \Bigg( \sum_{\nu, j \notin \mathcal{E}^{\nu}} \delta\eta_{j,\nu} X_{ext_{\nu,i}} \Bigg)^2 \gamma_j^2 + \sum_{i=1}^{N_y} \Bigg( 2 \sum_{\nu_1, j \notin \mathcal{E}^{\nu_1}} \eta_{j,\nu_1} X_{ext_{\nu_1,i}} \sum_{\nu_2, j \notin \mathcal{E}^{\nu_2}} \delta\eta_{j,\nu_2} X_{ext_{\nu_2,i}} \\
& + 2 \sum_{\mu, j \in \mathcal{E}^{\mu}} \hat{\eta}_{j,\mu} X_{ext_{\mu,i}} \sum_{\nu, j \notin \mathcal{E}^{\nu}} \delta\eta_{j,\nu} X_{ext_{\nu,i}} \Bigg) \gamma_j + \sum_{i=1}^{N_y} \Bigg( \Big( \sum_{\mu, j \in \mathcal{E}^{\mu}} (\hat{\eta}_{j,\mu}) X^{-1}_{ext_{\mu,i}} \Big)^2 + \Big( \sum_{\nu, j \notin \mathcal{E}^{\nu}} (\eta_{j,\nu}) X^{-1}_{ext_{\nu,i}} \Big)^2 \\
& + \Big( 2 \sum_{\mu, j \in \mathcal{E}^{\mu}} (\hat{\eta}_{j,\mu}) X^{-1}_{ext_{\mu,i}} \sum_{\nu, j \notin \mathcal{E}^{\nu}} (\eta_{j,\nu}) X^{-1}_{ext_{\nu,i}} \Big) \Bigg) - (U_{max_j})^2.
\end{aligned}
\tag{17}
$$

We solve for $\gamma$ using 17, choose a root that satisfies $0 \leq \gamma \leq 1$, and then find $\eta^{t+1}$ using 14.

## References


1. HC Wang, AM LeMessurier, DE Feldman, Tuning instability of non-columnar neurons in the salt-and-pepper whisker map in somatosensory cortex. *Nat. Commun.* **13** (2022).
2. A Ahmed, B Voelcker, S Peron, Representational drift in barrel cortex is receptive field dependent. *Curr. Biol.* **34** (2024).
3. JR Climer, H Davoudi, JY Oh, DA Dombeck, Hippocampal representations drift in stable multisensory environments. *Nature* (2025).
4. ME Rule, et al., Stable task information from an unstable neural population. *eLife* **9** (2020).
